# Supplementary material for: Environmental drivers of increased ecosystem respiration in a warming tundra
Source: Nature. 2024 Apr 17;629(8010):105–13. doi: 10.1038/s41586-024-07274-7 (PMC11062900; doi:10.1038/s41586-024-07274-7)
Supplement: Supplementary file 1 — Supplementary Methods, Discussion, Tables 1–5, Figs. 1–9 and references. [file 41586_2024_7274_MOESM1_ESM.pdf]

nature portfolio

https://doi.org/10.1038/s41586-024-07274-7

Supplementary information

Environmental drivers of increased ecosystem respiration in a warming tundra

In the format provided by the authors and unedited

Supplementary information

Supplementary (Supp.) Methods

Supp. Methods 1: Methods for the environmental drivers

To evaluate whether and how indirect warming effects and context-dependencies influenced the variation in the ecosystem respiration (*ER*) response to warming, we obtained information about several environmental drivers at the site, experiment, or *ER* measurement level (**Ext. Tables 3-4**), i.e. (i) drivers quantifying the change in environmental conditions due to warming (e.g. soil warming or changes in soil temperatures), as well as (ii) drivers that reflect context-specific environmental conditions irrespective of the warming treatment (e.g. ambient soil temperatures). Below is an overview per environmental driver on how we obtained it, categorized according to the type of driver, i.e. Climate, Soil, Vegetation, or Microbial.

(i) Indirect warming effects

We included drivers quantifying warming-induced changes in climatic and soil conditions and in vegetation and microbial community composition (**Ext. Table 3**). The drivers were included as Hedges SMD effect sizes based on (a) Climate, (b) Soil, (c) Vegetation and (d) Microbial data measured in control and warmed plots as detailed below. Note that not all drivers were available for all experiments or years, as this depended on whether or not they had been measured by data contributors (see **Supp. Table 5** for sample sizes).

(a) Climate: Air temperature, Soil temperature, and Soil moisture

Climatic drivers included air temperature (°C), soil temperature (°C), and soil moisture (%). Climate data were measured with environmental loggers alongside the *ER measurements*. Soil temperature and soil moisture data were submitted for different depths*, depending on data availability, ranging from 2-10cm. If data from multiple depths were available, we selected

the climate data from the depth closest to the most frequent depth across all datasets, i.e. 5cm.

**Overview of depths at which soil temperature and soil moisture data were provided:*

**Soil moisture**			**Soil temperature**		
**Depth (cm)**	**Number of datasets**	**Number of experiments**	**Depth (cm)**	**Number of datasets**	**Number of experiments**
2.5	5	5	2	7	6
3	11	5	2.5	1	1
3.5	9	5	3	2	1
3.75	3	1	**5**	**78**	**39**
**5**	**40**	**17**	7.5	8	4
6	13	1	10	22	5
7.5	11	5	**Total:**	**118**	**56**
10	19	5			
**Total:**	**111**	**44**			

(b) Soil: SOM, TC, TN, CN, BD, pH, and OLdepth

Soil drivers included soil organic matter (SOM, %), total C concentration (TC, %), total N concentration (TN, %), C:N-ratio, bulk density (BD, g cm^{-3}), and pH from the mineral and organic layer; as well as organic layer depth (OLdepth, cm). Soil data were measured at *plot* level in the same year as, or the closest year (either before or after) to, the *ER* measurements per dataset (see **Supp. Discussion 5** for more details on potential effects of differences in measurement years). Note that we refer to the soil total C and N concentration throughout the manuscript, since these drivers were measured on solid (dried) soil with CNS element analyzers and do therefore include both inorganic and organic compounds of carbon and nitrogen.

(c) Vegetation: Graminoids, Forbs, Deciduous shrubs, Evergreen shrubs, Mosses, and Lichen cover, aboveground Biomass, and Community height

Vegetation data included %cover per functional group (Graminoids, Forbs, Deciduous shrubs, Evergreen shrubs, Mosses, and Lichens), as well as aboveground biomass and mean height of the vegetation community at *plot* level. Cover percentages were based on field estimates

using the data-contributors' own protocols (e.g., pin-pointing, cover estimation). Aboveground biomass was based on actual biomass harvest data (19 datasets) as well as biomass estimated from vegetation community cover data (42 datasets). Height reflected mean community height based on field estimates (33 datasets followed our suggested protocol, 12 datasets followed their own protocol). Similarly as for the soil data, vegetation data were measured in the same year as, or the closest year in time to, the *ER* measurements per dataset (**Supp. Discussion 5**).

(d) Microbial community: Bacterial abundance, Fungal abundance, and FB-ratio

Microbial drivers included *plot*-level proxies for bacterial and fungal biomass and derived fungal-bacterial (FB)-ratios. These proxies were based on taxon-specific quantification of marker genes. All microbial data were collected according to a standardized protocol in 2013 in 8 of the experiments covered by our meta-analysis, which could be related to 16 *ER* measurement years (datasets). Details on the sampling process and microbial analyses can be found in Jeanbille et al. (2021)[1].

(ii) Context-dependencies

We collected information to compare the influence of variation in context-specific environmental conditions between the experiments on the *ER* response to warming in two different ways. First, similarly as for the indirect warming effects, we derived drivers from field measurements provided by data contributors, but we averaged climate and soil values from the control plots only here. Drivers quantifying context-specific environmental conditions related to the (a) Climate included Air temperature, Soil temperature and Soil moisture. Drivers related to the (b) Soil conditions included SOM, TC, TN, C:N-ratio, pH, BD, OLdepth (**Ext. Table 4, Fig. 4-5**). See details in *i) Indirect warming effects* above. Hence, these drivers comprised *plot*-specific data with a high level of detail, but at the same time they were not available from all experiments, lowering the sample size and power of these drivers in the meta-regressions. We supplemented these plot-specific drivers with map-derived values (e.g.

soil C stock) or categorical values (e.g. soil moisture class) at the *experiment* level. This was because we aimed to investigate context-dependencies across all experiments, and to also include context-dependencies related to the vegetation communities for which no ‘control’ measurements were available that were comparable across all experiments. Specifically, for (a) Climate, we added Zone and permafrost (PF- probability); for (b) Soil, we included soil C stock, soil moisture class, and soil pH class, and for (c) Vegetation, we included Vegetation Class, and Net Primary Productivity. As we did not want to mix these two data sources, there is some overlap for a few drivers reflecting similar conditions but with greater or smaller sample sizes and respectively coarser or more specific data quality (i.e. soil moisture and pH). See below for details. See **Supp. Table 5** for sample sizes for each of the context-dependent drivers.

(a) Climate: Zone, Permafrost (PF) probability

Zone

Data contributors classified their experiment into one of three bioclimatic zones: alpine, low Arctic or high arctic tundra. Alpine zones refer to tundra growing on altitudes >1000m, whereas low Arctic and high arctic zones refer to the bioclimatic subzones from the Circumpolar Arctic Vegetation Map D-E and A-C respectively[2].

Permafrost probability

For each experiment, we used a map-derived 1 km^2 value for probability of permafrost based on the map in Obu et al. (2019)[3].

(b) Soil: Soil C stock, Soil moisture class, Soil pH class

Soil C stock

We used a map-derived value for soil organic carbon (SOC) stock (tons ha^{-1}) at 0-30 cm soil depth at the experiment level. We combined two SOC sources, i.e. SOC_global, based on the global SOC map (see FAO & ITPS (2018)[4]) and SOC_circumpolar, based on the circumpolar soil map (see Hugelius et al. (2013)[5]). SOC_global was used as default and SOC_circumpolar

was used if the former was unavailable for the experiment. We preferred SOC_global over SOC_circumpolar because the former is more recent and more precise in terms of spatial accuracy.

Soil moisture class

We included soil moisture as a categorical driver, i.e. data contributors classified their experiment into one of three zones: dry, mesic, or wet. Soil moisture classes were broadly defined as wet (partly or continuously saturated, poor drainage, high water table), mesic (moist but not saturated, drains well, but never dries out), and dry (water drains or evaporates quickly leaving soils dry for most of the time).

Soil pH class

We additionally included soil pH as a categorical driver, i.e. data contributors classified their experiment into one of three pH classes: low (<5), medium (5-7), high (>7).

(c) Vegetation: Vegetation class, NPP

Vegetation class

For each experiment, we classified the vegetation into one of five broad categories of tundra systems (Barrens (B), Graminoid tundra (G), Prostrate shrub tundra (P), Erect shrub tundra (S), and Wetlands (W)). Data contributors classified their experiment into the most suitable Circumpolar Arctic Vegetation Map (CAVM) class[2]. For those experiments that were not included in the CAVM map (e.g. alpine tundra), we used a combination of data to determine the most comparable CAVM class, i.e. (i) broad description of the vegetation type by data contributors, (ii) vegetation cover of key functional groups, and (iii) soil moisture class.

NPP

For each site, we used a map-derived value of observed NPP (kg C m^{-2} $year^{-1}$) obtained through the MODIS satellite, see Running & Zhao (2019)[6].

(iii) Driver measurement years

Note that, in most cases, the vegetation, soil, and microbial data were measured in the same year or within 3 years from the *ER* measurements. For instance, 90% of the submitted '%Graminoids' driver data, 60% of 'TN', and 38% of 'FB_ratio' were measured in a year maximum 3 years different (before or after) the *ER* measurement year (90/100; 26/43; 6/16, respectively). See **Supp. Discussion 4** for the full overview of sample sizes, when applying different restrictive scenarios to limit the max. acceptable differences in years between *ER* and environmental driver data, and for how we assessed to what extent (mis-)matches between the measurement years of *ER versus* soil, vegetation, or microbial data might have influenced our results.

Supp. Methods 2: Methods for the upscaling

Flow diagram of the upscaling method: *Dark brown* boxes indicate the layers that were used, which were based on the variables that were included in the significant model to predict *ER* response. In *yellow*, the processing steps are indicate, i.e.: **1.** Aggregation of the layer products to mean for each 1 km x 1 km grid cell, **2.** Using information about the abundance of mineral layers for each grid cell, **3.** Weight averaging the products obtained in 1 and 2, Next, **4.**To estimate the uncertainty derived from the soil data, we used truncated multivariate random normal distribution generator to create 100 maps for both TN and C:N-ratio. Further, to include the uncertainty of the C:N-ratio estimates from SOC and TN instead of C:N-layer, we used measured values from our dataset, fitted 100 lines to it and used this to estimate the error derived from this assumption. Then, **5.** Using the ROM model from our study, we predicted the RE response to warming for each n = 100 set of TN and C:N-ratio map. Finally, **6.** The resulting n = 100 set of predicted values and associated standard errors were then combined to a mean and standard deviation for each grid cell. For a detailed description, see the *Methods*.

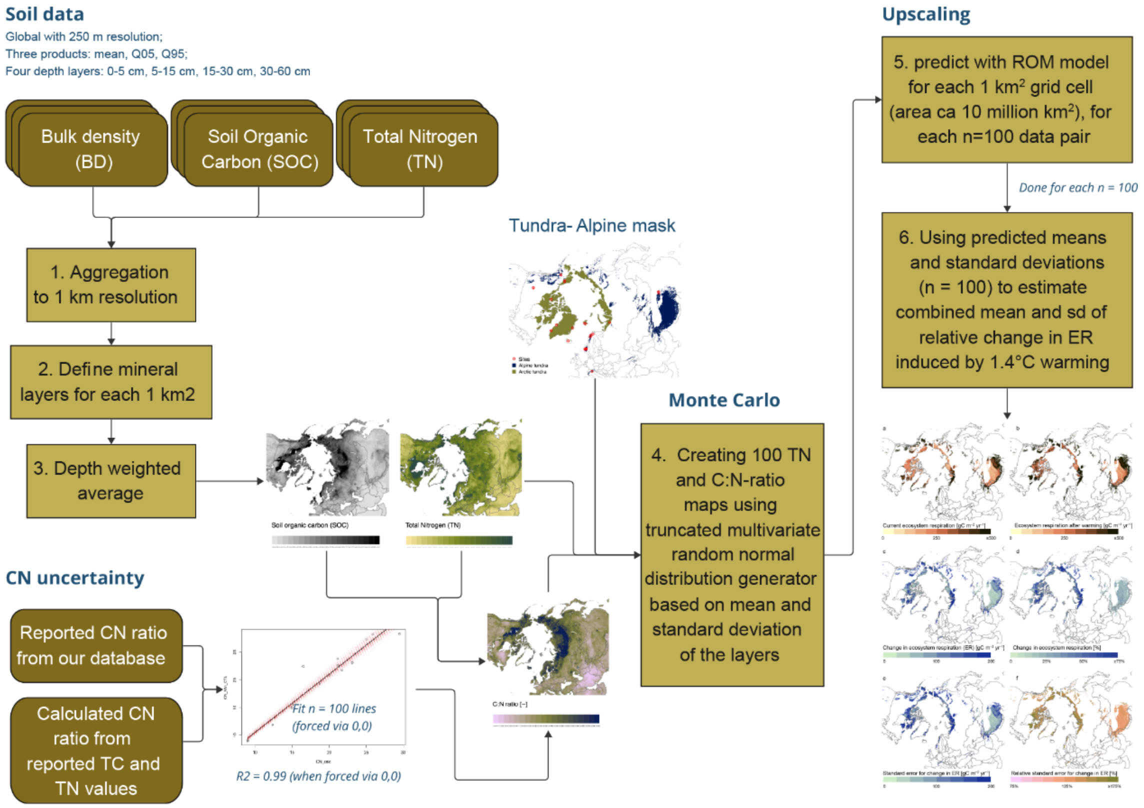

Supp. Discussions

In this section, we discuss the supplementary analyses that we performed to evaluate the robustness of our methodology and results, specifically related to quantifying the *ER* response to experimental warming with OTC chambers (methodology), and investigating the drivers of variation in the *ER* response (meta-regression results).

Supp. Discussion 1: *ER* partitioning: Autotrophic (*Ra*) vs. Heterotrophic respiration (*Rh*) response to warming

To understand whether the effect of warming on *ER* originated primarily from responses of plant or microbial respiration, we collected additional data from experiments used in our main analyses for which *ER* was partitioned into autotrophic (*Ra*) and heterotrophic respiration (*Rh*). *Ra* refers to the respiration of plants, roots and the associated rhizosphere (mycorrhizae and rhizosphere bacteria), while *Rh* refers to the respiration of soil micro-organisms that decompose dead organic materials[11]. For a subset of 9 out of our 136 *ER* datasets, covering 8 of the 56 experiments (see full overview below*), *Rh* was measured alongside *ER* in both the warmed and unmanipulated control plots. Each of these experiments used vegetation clipping to measure *Rh*. We calculated *Ra* by subtracting *Rh* from *ER*. We then calculated the effect sizes of mean growing season *Rh*, *Ra*, and *ER* response to warming (see overview below*), including Hedges' g Standardized Mean Difference (SMD) and the Ratio of Means (ROM) to be able to quantify percentage change in *ER*, *Rh*, and *Ra*, using the *escalc* function of the R package metafor. Next, we fitted random-effects meta-analysis models for each of the *ER*, *Rh*, and *Ra* effect sizes in a similar way as for the main *ER* dataset (see *Methods*) to test how *ER*, *Rh*, and *Ra* changed as a result of the warming treatment.

**Overview of the subset of experiments that measured heterotrophic respiration_(Rh) and autotrophic respiration (Ra) alongside-ecosystem respiration (ER), and the effect sizes used in the supplementary meta-analyses: Hedges SMD (left) and ROM (right).*

**Experiment**	**Year**	**Hedges SMD**			**ROM**		
		ER	***Rh***	***Ra***	***ER***	***Rh***	***Ra***
NOR_6	2020	1.49	0.93	1.09	0.55	0.36	0.67
NOR_8	2020	1.65	0.37	1.40	0.72	0.26	0.78
RUS_1	2013	0.31	0.71	-0.20	0.14	0.52	-0.19
RUS_2	2013	0.16	0.05	0.12	0.08	0.04	0.16
RUS_3	2013	0.72	0.62	0.00	0.27	0.48	0.00
SWE_14	2001	0.66	2.41	0.36	0.26	0.68	1.44
SWE_16	2006	0.58	0.78	0.21	0.34	0.43	0.34
SWE_16	2007	1.14	1.31	1.17	0.56	0.47	1.19
SWE_16	2011	1.41	2.33	0.90	0.66	0.41	1.54

The meta-analysis results (see **Ext. Table 1**) show two key findings. First, investigating *ER* responses to warming based on the subset of 9 datasets confirmed that the response was similar (in sign and magnitude) to the *ER* response based on the 136 datasets used in our main study (see **Ext. Table 1**), and we therefore assume that the *Ra* and *Rh* response to warming from the subset is representative for the full dataset as well. The increase in total ecosystem respiration (*ER*) observed in the smaller meta-analysis is similar to that in the larger dataset, although slightly higher: 39% compared to 30% (**Ext. Table 1**). The overlap within their confidence intervals, however, strongly suggests that the results are within a comparable range. Second, the strong increase in *ER* with warming based on the subset of 9 datasets, appeared driven by increased plant-related and heterotrophic respiration, as both show strongly positive Hedges SMD and ROM as well.

Supp. Discussion 2: Warming and *ER* measurement methodology

Despite the shared experimental warming-setup, experiments differed in OTC height (0.3-0.7m height); seasonal removal of OTCs (e.g. year-round placement or winter removal of OTCs); machine type for CO_2 measurements (infrared (IR) or laser);

measurement system (automated or manual and closed or open); analyzer producer (Los Gatos Research (LGR), LICOR, PP-system, or Vaisala), flux measurement plot size, and timing of measurement (night-time vs. daytime *ER*). We therefore tested whether these methodological differences influenced the *ER* response to warming. Thereto, we ran a multi-factor meta-regression model including the factors 'OTC height', 'Plot size', 'OTC removal', 'Machine type', 'Measurement system', and 'Timing'. We did not include 'Analyzer producer' because this information was strongly related to the '*Machine Type'*. The final model was backward selected by comparing AIC values of all possible factor combinations, and showed no significant effects (p>0.05) of any of the drivers. We also ran single-factor models including all these drivers separately, for which we also did not find significant effects of any of the drivers. We therefore conclude that the *ER* response was not affected by measurement methodology, and did not include these factors in our further meta-analysis or -regression models as confounding factor.

Supp. Discussion 3: Potential interaction of OTC microclimate effects

Surprisingly, we did not find that changes in the microclimate caused by the OTCs (i.e., higher air and soil temperature, lower soil moisture; **Supp. Table 3**) were related to the *ER* response (**Ext. Table 3**). Because potential stimulating effects of higher soil temperatures on *ER* might have been masked by potential negative effects of soil drying occurring at the same time, we tested the interaction between soil temperature and moisture on the *ER* response with a two-factor meta-regression model (mods = ~ soil temp * soil moist). This model did not show a significant main or interactive effect, and we therefore conclude that there is no interaction masking possible microclimatic effects on the *ER* response.

Supp. Discussion 4: Sensitivity analysis meta-regressions for 'indirect warming effects' and 'context-dependencies'

To analyze relationships between a range of potential environmental drivers that represented indirect warming effects and context-dependencies, and the *ER* responses, we used plot-level field measurements reflecting soil, vegetation and microbial community conditions from the same experiments as the *ER* data, provided by the data contributors (only where these were available, see **Supp. Table 2**). The remoteness of many of these tundra field experiments often hampers regular, manual measurements in the field. In addition, the measurement of some environmental drivers is too labor intensive, destructive or specialized to be performed repeatedly with high frequency across all experiments. Data contributors were thus asked to provide as many environmental driver data as they had available, and to match these as closely in time as possible with their *ER* data. Still, our environmental data on the soil, vegetation, and microbial community drivers had the following characteristics that could have influenced the meta-regression results: 1) driver data were not always available for the same year as the *ER* data (i.e. inducing differences in years between *ER* and driver data), and 2) they were not always available from multiple years per experiment (i.e. inducing replicated driver data). Data contributors were asked to match their environmental driver data as closely in time as possible with their *ER* data. These characteristics led to case-specific scenarios, depending on data availability per site, with some experiments having more accurately temporally matched *ER*-driver data than others, e.g. 3 years of *ER* data coupled to 3 years of a measured driver, vs. 3 years of *ER* data coupled to 1 (same) year of a measured driver.

We performed a sensitivity analysis to see how this case-specific matching in years between *ER* and environmental driver data might have affected our meta-regression results. We therefore re-ran all meta-regression models with $p<0.1$ with different scenarios to increasingly restrict our sample sizes related to the two characteristics that might have caused bias, i.e. difference in calendar years between *ER* and drivers, and replication of drivers to be coupled to multiple *ER* datasets (years). We then compared the model output (i.e. QM, slope (beta), *p*-value, and sample size) across these scenarios with the output from the full or unrestricted model (**Supp. Figure 4**). The 7 restrictive scenarios included: A) differences in calendar year between the *ER* and the driver is maximum 0 (i.e., measured in exactly same year), 1, 2, 3, 4, or 5 years difference; and B) driver data cannot be replicated, and are only coupled to the *ER* year closest to the driver year. See below* for the overview of the number of datasets per environmental soil, vegetation, and microbial driver in the 8 sample size scenarios (7 restrictive + 1 unrestrictive).

This sensitivity analysis showed that overall, the slopes of the model (Beta) did not appear strongly influenced by the sample size (restriction), especially for most of the drivers that are significant in the full (unrestricted) scenario and reported and presented in the main text (**Supp. Figure 4b**). This suggests that the direction of the relations between drivers and *ER* response to warming are robust. That is, not affected by the discrepancy between measurement years of *ER* and driver data or by replication. On the other hand, QM values, i.e. the 'importance of moderator' reflecting the power of a driver in explaining the *ER* response, as well as the *p*-values were strongly driven by the sample size (restriction), where QM was higher, and *p*-values more significant for the fullest model for most of the drivers that are significant in the

full (unrestricted) scenario. This implies that the models were less powerful to detect effects due to decreasing the sample sizes in more restrictive sample size scenarios. We therefore conclude that using restrictive scenarios would compromise the power to detect relations in our database from field-based tundra warming experiments too strongly, including multiple labor intensive, destructive or specialized driver measurements. At the same time, using the full (unrestricted) database for the meta-regressions does not appear to influence the relations (Beta) for the significant drivers. We therefore deem the meta-regression results based on the full database the best, most realistic representation we were able to get from field measurements taken in the actual tundra warming experiments under study and therefore reported and interpret these in the main text.

**Overview of the number of datasets per environmental soil, vegetation, and microbial driver in 7 restrictive sample size scenarios and the full model. From left to right, scenario of no replication of driver measurements, i.e. only including driver data once and for the ER year closest to the driver year (‘No replication’), scenarios where calendar year differences between ER measurements and driver measurements are restricted to maximum 0 to 5 years (‘Max 0-5 years diff’); and ‘All data’, i.e. scenario including all data without restrictions based on driver measurement years.*

Analysis	Type	Driver	No replication	Max 0 year diff	Max 1 year diff	Max 2 years diff	Max 3 years diff	Max 4 years diff	Max 5 years diff	All data
**Indirect warming effects**	**Soil**	SMD_SOM_Org	22	20	24	26	26	26	26	41
		SMD_SOM_Min	12	12	13	13	13	13	13	28
		SMD_TC_Org	23	21	36	44	46	50	50	65
		SMD_TC_Min	12	12	18	21	24	27	27	42
		SMD_TN_Org	28	21	36	44	46	50	50	65
		SMD_TN_Min	13	12	18	21	24	27	27	42
		SMD_CN_Org	32	26	41	50	52	56	56	71
		SMD_CN_Min	12	11	17	19	21	23	23	38
		SMD_pH_Org	29	23	30	34	35	38	38	53
		SMD_pH_Min	10	9	10	10	11	12	12	27
		SMD_BD_Org	18	15	22	29	32	34	34	49
		SMD_BD_Min	8	8	12	14	15	16	16	31
		SMD_OL_D_Plot	18	17	22	27	27	27	27	44
	**Vegetation**	SMD_Graminoids	26	48	69	79	90	91	92	100
		SMD_Forbs	72	52	77	90	101	102	103	111
		SMD_Decid_Shrubs	60	39	57	69	80	81	82	90
		SMD_Evergr_Shrubs	53	33	55	67	78	79	80	88
		SMD_Mosses	59	47	66	75	82	83	84	88
		SMD_Lichens	50	41	57	65	72	73	74	78
		SMD_BM	28	21	33	48	54	55	55	61
		SMD_Mean_H	25	14	21	28	36	36	36	43
	**Microbial**	SMD_Bacterial_BM_Weight	8	1	3	6	6	6	8	16
		SMD_Fungal_BM_Weight	8	1	3	6	6	6	8	16
		SMD_FB_Ratio	8	1	3	6	6	6	8	16
**Context-dependencies**	**Soil**	SOM_Org_CTL	23	21	24	26	26	26	26	41
		SOM_Min_CTL	12	12	13	13	13	13	13	28
		TC_Org_CTL	29	24	42	51	52	55	55	70
		TC_Min_CTL	14	13	20	22	24	26	26	42
		TN_Org_CTL	33	23	41	51	52	55	55	70
		TN_Min_CTL	14	13	20	24	26	28	28	43
		CN_Org_CTL	36	28	46	56	57	60	60	75
		CN_Min_CTL	13	12	19	22	23	24	24	39
		pH_Org_CTL	31	23	32	36	37	40	40	55
		pH_Min_CTL	11	10	12	12	13	14	14	29
		BD_Org_CTL	28	21	34	47	51	54	54	69
		BD_Min_CTL	11	10	15	19	21	23	23	38
		OL_D_Plot_CTL	34	31	42	52	54	55	56	78

Supp. Discussion 5: Unbalanced *ER* time series across experiments

ER data from 56 independent OTC experiments was used in the analyses, where each experiment contributed between 1 and 13 measurement years of *ER* data (**Supp. Table 2, Supp. Fig. 1**), and between one and multiple measurement occasions each year (**Supp. Fig 2**). Using seasonally averaged *ER* data based on different measurement occasions, and using different number of datasets (*ER* measurement years) per experiment across the analysis implies different precision in the data across the experiments.

To accommodate for differences in precision related to the number of *ER* measurements across the growing season, we calculated Hedges' g Standardized Mean Difference (SMD), which is an effect size that divides the differences in means of both comparison groups (control and OTC) by the pooled standard deviation. Hence, it is accompanied by a confidence interval that reflects the precision of the effect size for each dataset. In this way, datasets that were more precise, i.e. based on more measurements per season, weighed more in the meta-analyses and -regressions.

To accommodate for differences in precision related to the number of *ER* years contributed per experiment, we repeated our meta-analysis on a subset excluding the data from two 'outlier' experiments. The experiments ALA_1 and GRE_6 contributed data from many more measurement occasions per season (16273 and 1333 resp.) and/or from more years (11 and 13 years) than other experiments and therefore may have weighed disproportionally strong on the results. The results were similar as for the full dataset: mean pooled effect size of 0.59 (Hedges SMD, 95% CI [0.47, 0.72], N=112, p <0.001) as when all experiments and years were included: mean pooled effect size of 0.57 (95% CI [0.44-0.70], N=136, p<0.001).

Supp. Discussion 6: Temporal patterns in *ER* response to warming *within* experiments

Here, we investigated *within*-experiment temporal patterns in the variation of the *ER* response to warming, in addition to the temporal patterns observed *across* experiments that we reported in the main text (**Fig. 3**). Thereto, we extracted the slopes of linear regression models testing the effect of warming duration on the *ER* response for each experiment, both (a) across and (b) within age classes. Then, we performed meta-analyses testing whether overall the slope was significantly different from zero, which would indicate significant overall temporal trends within experiments. The results are presented in the table below*. We visualized these linear regressions in **Supp. Fig. 5.**

**Results of the meta-analysis models on experiment-specific linear regression slopes of ER Hedges SMD~Duration across (a) and within (b) age classes.*

Response	Slope [95% CI]	Q-value	tau^2	tau	I^2 in %	H^2	N
(a) Across age classes							
**NA**	**0.52 [0.01, 0.09] ***	**69**	**0.005**	**0.067**	**48.2**	**1.9**	**34**
(b) Within age classes							
[0-5) years	-0.03 [-0.14, 0.09] ns	16	0	0	0	1	18
**[5-10) years**	0.13 [-0.03, 0.29] ns	83	0.047	0.216	93.4	15.2	8
**[10-15) years**	**0.31 [0.08, 0.54] ****	**2**	**0.014**	**0.118**	**48.6**	**1.9**	**2**
≥ 15 years	0.02 [-0.22, 0.25] ns	1	0	0	0	1	4

[1]Significance levels: *** <0.05; ** <0.01. Significant results with *p*<0.05 are highlighted in bold.**

The results (a) across age classes showed a significant overall effect, with mean slope of 0.52, suggesting that the *ER* response to warming increased with warming duration when evaluating *within*-experiment patterns. As a comparison, when evaluating temporal patterns *across* experiments and across age classes (reported in the main text), we found no significant effect. The results (b) within age classes showed no significant relation of *ER* response with warming duration in age classes [0-5), [5-10),

and ≥ 15 years, while a the *ER* response significantly increased with warming duration in age class [10-15) years. As a comparison, when evaluating the temporal patterns *across* experiments (main text), we found similar results, except for a significant decreasing trend in age class [5-10) years, which is not found here *within* experiments.

It should be noted that these within-experiment temporal analyses, especially within age classes and for longer warming duration, are based on very small sample sizes (see table* above). Moreover, there are currently only two experiments with long time series of 11 and 13 years of subsequent *ER* data (ALA_1, GRE_6), thus covering multiple age classes, while all other experiments provided 1-4 measurement years of *ER* data only (see table below*), often covering only single age classes. While the above *within*-experiment analyses thus strengthen rather than weaken our conclusion that experimental warming causes a continued increase in *ER*, we argue that the analyses *across* experiments, as reported in the main text, are more reliable representations of the overall the temporal patterns of *ER* responses to warming. We have therefore based our conclusions using exclusively the across-experiment temporal analyses.

**Overview of the variability in time series length of ER years (i.e. Nr of repeated ER measurement years) within an experiment.*

**Nr of *ER* years**	**Nr of experiments**
1	21
2	16
3	9
4	8
11	1
13	1

Supp. Tables

Supp. Table 1: General information on the datasets used in the meta-analysis: 136 datasets from 56 warming experiments covering 28 sites.

Country	Site	Exp ID	Location (DD)	Zone	PFprob	Soil Moisture class	pH class	Soil C stock (tons ha-1)	Vegetation class	NPP (kg C m-2 yr-1)	*ER* years	Nr *ER* Years	Warming duration
Alaska (USA)	Eight Mile Lake	ALA_1	63.88, -149.23	low Arctic	0.50	mesic	Medium	177.00	G	0.28	2009-2019	11	1-11
	Toolik	ALA_2	68.63, -149.58	low Arctic	1.00	wet	Low	128.00	G	0.18	2015	1	0
		ALA_3	68.63, -149.58	low Arctic	1.00	wet	Low	128.00	G	0.18	2016	1	0
	Ice Cut	ALA_4	69.05, -148.84	low Arctic	1.00	mesic	Medium	128.00	G	0.20	2017, 2019	2	1, 3
Australia	Silver Plains	AUS_1	-42.08, 147.09	Alpine	NA	wet	Medium	117.73	G	0.77	2016-2018	3	2-4
Canada	Wandering River	CAN_2	55.36, -112.52	low Arctic	0.08	wet	Low	110.99	W	0.52	2011-2013	3	0-2
		CAN_3	55.35, -112.52	low Arctic	0.08	mesic	Low	110.99	W	0.52	2011-2013	3	0-2
		CAN_4	55.28, -112.47	low Arctic	0.00	dry	Low	109.56	W	0.48	2011-2013	3	0-2
	Cambridge Bay	CAN_5	69.13, -105.06	high Arctic	1.00	mesic	High	121.00	P	0.06	2018-2019	2	6-7
China	Haibei	CHI_1	37.62, 101.18	Alpine	0.00	mesic	High	88.13	G	0.34	2019-2020	2	5-6
		CHI_2	37.69, 101.36	Alpine	0.99	mesic	High	91.31	G	0.32	2019-2020	2	4-5
		CHI_3	37.71, 101.37	Alpine	1.00	mesic	High	55.41	G	0.18	2019-2020	2	5-6
	Gangca	CHI_4	37.75, 100.08	Alpine	1.00	wet	Medium	71.96	G	0.24	2014-2016	3	1-3
Finland	Kilpisjärvi	FIN_1	69.07, 20.82	low Arctic	0.08	dry	Low	44.49	S	0.16	2013	1	19
Greenland	Blaesedalen	GRE_1	69.31, -53.51	low Arctic	0.94	dry	Medium	97.00	S	0.04	2013-2014	2	1-2
		GRE_2	69.31, -53.51	low Arctic	0.94	wet	High	97.00	W	0.04	2014	1	1
	Zackenberg	GRE_4	74.47, -20.53	high Arctic	1.00	dry	High	97.00	P	0.04	2012, 2015	2	5, 8
		GRE_5	74.47, -20.53	high Arctic	1.00	dry	High	97.00	P	0.04	2012, 2015	2	5, 8
	Kobbefjord	GRE_6	64.14, -51.38	low Arctic	0.37	mesic	Medium	57.00	S	0.13	2008-2020	13	1-13
Iceland	Audkuluheidi	ICE_1	65.22, -19.7	low Arctic	0.00	mesic	Medium	126.67	S	0.08	2007-2008	2	10-11
Italy	Gavia_Valley	ITA_1	46.34, 10.5	Alpine	0.34	mesic	Low	NA	B	0.10	2020	1	12
		ITA_2	46.34, 10.5	Alpine	0.34	mesic	Low	NA	S	0.10	2020	1	12
		ITA_3	46.34, 10.5	Alpine	0.34	mesic	Low	NA	B	0.10	2020	1	8
		ITA_4	46.34, 10.5	Alpine	0.34	mesic	Low	NA	S	0.10	2020	1	8
		ITA_5	46.35, 10.49	Alpine	0.47	mesic	Low	NA	G	0.26	2020	1	2
Norway	*R*aisduoddar	NOR_1	69.31, 21.19	low Arctic	0.38	mesic	Medium	133.81	S	0.04	2011-2012	2	1-2

	Iskoras	NOR_6	69.34, 25.3	low Arctic	0.05	mesic	Low	185.50	S	0.20	2020	1	3
		NOR_7	69.34, 25.3	low Arctic	0.05	mesic	Low	185.50	S	0.20	2020	1	3
		NOR_8	69.34, 25.3	low Arctic	0.05	wet	Low	185.50	S	0.20	2020	1	3
	Lavisdalen	NOR_9	60.82, 7.28	Alpine	0.00	mesic	Medium	60.30	G	0.16	2020	1	2
	Ulvhaugen	NOR_10	61.02, 8.12	Alpine	0.01	mesic	Medium	93.14	G	0.16	2020	1	2
	Gudmesdalen	NOR_11	60.83, 7.18	Alpine	0.00	mesic	Medium	60.30	G	0.23	2020	1	2
	Skjellingahaugen	NOR_12	60.93, 6.42	Alpine	0.00	mesic	Medium	93.14	G	0.13	2020	1	2
Russia	Seida	RUS_1	67.07, 62.93	low Arctic	0.41	dry	Medium	156.60	S	0.18	2012-2014	3	0-2
		RUS_2	67.06, 62.93	low Arctic	0.41	dry	Low	110.60	S	0.18	2012-2014	3	0-2
		RUS_3	67.06, 62.93	low Arctic	0.41	mesic	Low	110.60	B	0.18	2012-2014	3	0-2
Svalbard (Norway)	Adventdalen	SVA_1	78.17, 16.04	high Arctic	0.94	mesic	Medium	17.00	P	0.02	2016-2017	2	0-1
		SVA_2	78.17, 16.04	high Arctic	0.94	mesic	Medium	17.00	P	0.02	2016-2017	2	0-1
		SVA_3	78.17, 16.04	high Arctic	0.94	wet	Medium	17.00	W	0.02	2016-2017	2	0-1
		SVA_4	78.17, 16.02	high Arctic	0.94	mesic	Medium	17.00	P	0.03	2018-2019	2	2-2
		SVA_5	78.16, 16.1	high Arctic	0.97	wet	Medium	17.00	W	0.02	2003-2005, 2007	4	0-2, 4
		SVA_6	78.16, 16.1	high Arctic	0.97	dry	Medium	17.00	G	0.02	2003-2005, 2007	4	0-2, 4
	Endalen	SVA_7	78.19, 15.76	high Arctic	0.97	dry	Medium	17.00	P	0.02	2018	1	16
		SVA_8	78.19, 15.76	high Arctic	0.97	mesic	Medium	17.00	P	0.02	2018	1	16
		SVA_9	78.19, 15.76	high Arctic	0.97	mesic	Medium	17.00	B	0.02	2018	1	16
Sweden	Latnjajaure	SWE_1	68.35, 18.49	low Arctic	0.17	wet	Medium	122.00	G	0.08	2008, 2017-2019	4	13, 22-24
		SWE_2	68.36, 18.49	low Arctic	0.17	dry	Medium	122.00	P	0.10	2008, 2017-2019	4	13, 22-24
		SWE_3	68.36, 18.5	low Arctic	0.17	mesic	Medium	122.00	G	0.10	2008, 2017-2019	4	14, 23-25
		SWE_4	68.36, 18.5	low Arctic	0.17	dry	Medium	122.00	P	0.10	2008, 2017-2019	4	14, 23-25
		SWE_5	68.36, 18.5	low Arctic	0.17	wet	Medium	122.00	W	0.10	2008, 2017-2019	4	14, 23-25
	Multe	SWE_6	68.35, 18.83	low Arctic	0.00	mesic	High	122.00	S	0.22	2006-2007, 2015	3	7, 8, 16
	Paddus_1	SWE_7	68.33, 18.85	low Arctic	0.00	dry	High	122.00	S	0.17	2011	1	22
	Paddus_2	SWE_12	68.31, 18.86	low Arctic	0.00	mesic	Low	122.00	P	0.16	2001, 2018	2	3, 20
		SWE_14	68.32, 18.84	low Arctic	0.01	mesic	Low	122.00	P	0.17	2001	1	3
		SWE_15	68.33, 18.84	low Arctic	0.00	mesic	Medium	122.00	S	0.17	2001, 2018	2	3, 20
	Abisko	SWE_16	68.35, 18.82	low Arctic	0.01	wet	Low	122.00	S	0.24	2003, 2005-2007	4	3, 5-7
**12 countries**	**28 sites**	**56 experiments**									**136 *ER* measurement years or datasets**		

General information on the datasets included in the meta-analysis. From left to right: country ('Country') and site ('Site') where the warming experiment is located; unique identification code of the warming experiment ('Exp ID') with the three first letters representing the country code; location in decimal degrees of the experiment ('Location'); environmental conditions quantifying the climate (climate zone or 'Zone', permafrost probability or 'PFprob'), the soil ('Soil Moisture class', 'pH class', and 'Soil C stock'), and vegetation ('Vegetation class', 'NPP'); specific *ER* measurement years as well as the number of years per experiment ('*ER* years', 'Nr *ER* years'); and the experimental warming duration in years at the time of *ER* measurements ('Warming duration'). See **Supp. Methods 1** for details on the environmental drivers. Countries with multiple Sites are highlighted with a grey background, as well as Sites with multiple Experiments (i.e. reflecting different vegetation communities or site conditions), and Experiments with multiple *ER* measurement years (i.e. 'Datasets' throughout the manuscript)

Supp. Table 2: Sample sizes (number of *ER* observations and plots) and weights for the 136 *ER* datasets used in the meta-analysis.

Nr	DS (Exp_ID_Year)	Tot Obs	Day Obs	Tot CTL Plots	Tot OTC Plots	Weights
1	ALA_1_2009	1673	80	12	12	2.3
2	ALA_1_2010	1894	92	12	12	4.0
3	ALA_1_2011	2576	92	12	12	4.1
4	ALA_1_2012	1496	91	12	12	3.8
5	ALA_1_2013	1563	92	12	12	3.8
6	ALA_1_2014	1532	92	12	12	3.8
7	ALA_1_2015	1453	92	12	12	3.7
8	ALA_1_2016	1271	90	12	12	3.6
9	ALA_1_2017	1324	90	12	12	3.6
10	ALA_1_2018	1101	91	12	12	3.2
11	ALA_1_2019	390	91	2	1	1.7
12	ALA_2_2015	138	6	12	12	0.3
13	ALA_3_2016	35	6	3	3	0.2
14	ALA_4_2017	43	6	12	12	0.4
15	ALA_4_2019	141	15	12	12	0.6
16	AUS_1_2016	31	2	9	8	0.3
17	AUS_1_2017	16	1	8	8	0.2
18	AUS_1_2018	32	2	8	8	0.3
19	CAN_2_2011	87	11	6	6	0.8
20	CAN_2_2012	143	12	6	6	1.1
21	CAN_2_2013	127	9	6	6	0.9
22	CAN_3_2011	69	9	6	6	0.7
23	CAN_3_2012	139	12	6	6	1.1
24	CAN_3_2013	116	9	6	6	0.9
25	CAN_4_2011	76	9	6	6	0.7
26	CAN_4_2012	153	10	6	6	1.1
27	CAN_4_2013	105	7	6	6	0.9
28	CAN_5_2018	16	3	4	4	0.2
29	CAN_5_2019	26	5	5	5	0.3
30	CHI_1_2019	24	3	4	4	0.3
31	CHI_1_2020	24	3	4	4	0.3
32	CHI_2_2019	16	2	4	4	0.2
33	CHI_2_2020	24	3	4	4	0.2
34	CHI_3_2019	16	2	4	4	0.2
35	CHI_3_2020	24	3	4	4	0.2
36	CHI_4_2014	158	8	10	10	1.1
37	CHI_4_2015	159	8	10	10	1.4
38	CHI_4_2016	178	9	10	10	1.1
39	FIN_1_2013	69	11	5	5	0.3
40	GRE_1_2013	103	9	6	6	0.8
41	GRE_1_2014	128	12	6	6	0.8

42	GRE_2_2014	81	7	6	6	0.3
43	GRE_4_2012	33	4	5	5	0.3
44	GRE_4_2015	38	4	5	5	0.3
45	GRE_5_2012	50	5	5	5	0.4
46	GRE_5_2015	34	4	5	5	0.3
47	GRE_6_2008	129	12	6	6	0.9
48	GRE_6_2009	120	11	6	6	1.2
49	GRE_6_2010	137	12	6	6	1.3
50	GRE_6_2011	96	9	6	6	1.0
51	GRE_6_2012	135	12	6	6	1.3
52	GRE_6_2013	119	13	6	6	1.2
53	GRE_6_2014	122	11	6	6	1.2
54	GRE_6_2015	96	9	6	6	1.1
55	GRE_6_2016	143	13	6	6	1.3
56	GRE_6_2017	114	11	6	6	1.2
57	GRE_6_2018	122	11	6	6	1.2
58	GRE_6_2019	113	10	6	6	1.1
59	GRE_6_2020	104	9	6	6	0.9
60	ICE_1_2007	12	1	6	6	0.2
61	ICE_1_2008	36	3	6	6	0.3
62	ITA_1_2020	40	4	5	5	0.2
63	ITA_2_2020	40	4	5	5	0.2
64	ITA_3_2020	18	3	3	3	0.2
65	ITA_4_2020	17	3	3	3	0.2
66	ITA_5_2020	30	3	5	5	0.2
67	NOR_1_2011	198	13	8	8	0.9
68	NOR_1_2012	102	7	8	8	0.9
69	NOR_6_2020	32	5	6	6	0.2
70	NOR_7_2020	26	4	8	6	0.2
71	NOR_8_2020	24	5	5	5	0.2
72	NOR_9_2020	28	3	5	5	0.2
73	NOR_10_2020	29	3	5	5	0.2
74	NOR_11_2020	30	3	5	5	0.2
75	NOR_12_2020	25	4	5	5	0.2
76	RUS_1_2012	69	7	5	5	0.6
77	RUS_1_2013	90	9	5	5	0.7
78	RUS_1_2014	20	2	5	5	0.3
79	RUS_2_2012	57	6	5	5	0.6
80	RUS_2_2013	90	9	5	5	0.7
81	RUS_2_2014	17	2	5	5	0.3
82	RUS_3_2012	57	6	5	5	0.5
83	RUS_3_2013	87	9	5	5	0.6
84	RUS_3_2014	12	2	4	5	0.2
85	SVA_1_2016	77	21	14	14	0.6
86	SVA_1_2017	81	20	14	14	0.6
87	SVA_2_2016	80	19	14	14	0.6

88	SVA_2_2017	83	20	14	14	0.6
89	SVA_3_2016	70	18	12	12	0.6
90	SVA_3_2017	71	18	12	12	0.6
91	SVA_4_2018	36	2	9	9	0.3
92	SVA_4_2019	35	2	9	9	0.3
93	SVA_5_2003	18	2	5	5	0.2
94	SVA_5_2004	30	3	5	5	0.4
95	SVA_5_2005	50	17	5	5	0.5
96	SVA_5_2007	30	3	5	5	0.3
97	SVA_6_2003	20	2	5	5	0.3
98	SVA_6_2004	30	3	5	5	0.4
99	SVA_6_2005	49	16	5	5	0.5
100	SVA_6_2007	40	5	5	5	0.4
101	SVA_7_2018	18	2	5	5	0.2
102	SVA_8_2018	10	2	3	3	0.1
103	SVA_9_2018	17	2	5	5	0.2
104	SWE_1_2008	50	5	5	5	0.3
105	SWE_1_2017	50	6	5	5	0.5
106	SWE_1_2018	59	6	5	5	0.5
107	SWE_1_2019	10	2	5	5	0.1
108	SWE_2_2008	32	4	4	4	0.2
109	SWE_2_2017	48	6	4	4	0.4
110	SWE_2_2018	40	5	4	4	0.3
111	SWE_2_2019	8	2	4	4	0.1
112	SWE_3_2008	49	5	5	5	0.3
113	SWE_3_2017	60	6	5	5	0.5
114	SWE_3_2018	50	5	5	5	0.5
115	SWE_3_2019	10	2	5	5	0.1
116	SWE_4_2008	49	5	5	5	0.3
117	SWE_4_2017	44	6	5	5	0.4
118	SWE_4_2018	50	5	5	5	0.4
119	SWE_4_2019	10	2	5	5	0.1
120	SWE_5_2008	35	5	5	5	0.3
121	SWE_5_2017	30	4	5	5	0.4
122	SWE_5_2018	59	6	5	5	0.5
123	SWE_5_2019	8	1	4	4	0.1
124	SWE_6_2006	36	4	6	6	0.3
125	SWE_6_2007	44	4	6	6	0.4
126	SWE_6_2015	84	7	6	6	0.3
127	SWE_7_2011	156	13	6	6	0.3
128	SWE_12_2001	70	6	5	5	0.3
129	SWE_12_2018	54	6	8	8	0.3
130	SWE_14_2001	60	6	5	5	0.3
131	SWE_15_2001	30	2	5	5	0.2
132	SWE_15_2018	47	6	7	7	0.3
133	SWE_16_2003	37	4	5	5	0.4

134	SWE_16_2005	50	5	5	5	0.6
135	SWE_16_2006	70	7	5	5	0.7
136	SWE_16_2007	40	4	5	5	0.4

From left to right, the columns show the dataset number (Nr), an alphabetically + chronologically ordered number per dataset (‘DS’) consisting of the unique experimental code and the year of flux measurement (Exp_ID_Year’), the total number of *ER* measurements or observations available for each dataset (‘Tot Obs’), the number of day-observations used for each dataset (‘Day Obs’), the number of OTC and control plots where *ER* measurements were taken (‘OTC Plots’, ‘CTL Plots’), and the weights per dataset as used in the main meta-analysis.

Supp. Table 3: Effects of OTC warming treatment on local environmental conditions.

Type	Driver	Slope [95%CI] [1]	Driver	Slope [95%CI] [1]
	Effect size:	Hedges SMD		Raw mean difference (RMD)
**Micro-climate**	***Air temperature***	**↑ 0.30 [0.18, 0.43] *****	***Air temperature***	**↑ 1.44 [0.85, 2.03]°C *****
	Soil temperature	**↑ 0.18 [0.08, 0.29] *****	***Soil temperature***	**↑ 0.44 [0.24, 0.65]°C *****
	Soil moisture	**↓ −0.40 [−0.55, −0.25] *****	***Soil moisture***	**↓ -1.58 [-2.35, -0.82]% *****
**Soil conditions**	***SOM*** *(**mineral**, organic)*	**↑ 0.36 [0.02, 0.71] *** ↓ -0.12 [-0.39, 0.13]	*SOM (mineral, organic)*	↑ 1.23 [-0.18, 2.65]% (*) ↓ -0.31 [-0.65, 0.04]% (*)
	TC (mineral, organic)	↑ 0.26 [-0.10, 0.61] ↓ -0.06 [-0.29, 0.18]	*TC (mineral, organic)*	↑ 0.23 [-0.13, 0.60]% ↓ -0.01 [-0.22, 0.21]%
	TN (mineral, organic)	↑ 0.28 [-0.08, 0.63] ↓ -0.09 [-0.32, 0.15]	***TN*** *(mineral, **organic**)*	↑ 0.02 [-0.01, 0.05]% **↓ -0.01 [-0.02, 0.00]% ****
	CN (mineral, organic)	↓ -0.16 [-0.50, 0.19] ↑ 0.09 [-0.12, 0.29]	***CN*** *(mineral, **organic**)*	↓ -0.95 [-2.72, 0.83] **↑ 1.11 [0.12, 2.10] ***
	pH (mineral, organic)	↓ -0.27 [-0.64, 0.10] ↓ -0.14 [-0.34, 0.06]	***pH*** *(mineral, **organic**)*	↓ -0.03 [-0.08, 0.01] **↓ -0.12 [-0.20, -0.03] ****
	Bulk density (mineral, organic)	↓ -0.24 [-0.70, 0.22] ↓ -0.16 [-0.44, 0.12]	*Bulk density (mineral, organic)*	↓ -0.08 [-0.15, 0.00] g cm^{-3} (*) 0.00 [-0.01, 0.00]g cm^{-3}
	Organic layer depth	↑ 0.17 [-0.11, 0.44]	*Organic layer depth*	↑ 0.14 [-0.03, 0.31]cm (*)
**Vegetation community**	*Graminoids*	↑ 0.06 [-0.10, 0.21]	*Graminoids*	NA
	Forbs	0.00 [-0.12, 0.12]	*Forbs*	NA
	Deciduous shrubs	↑ 0.02 [-0.18, 0.22]	*Deciduous shrubs*	NA
	Evergreen shrubs	↑ 0.06 [-0.14, 0.27]	*Evergreen shrubs*	NA
	Mosses	↑ 0.03 [-0.25, 0.31]	*Mosses*	NA
	Lichens	**↓ -0.21 [-0.35, -0.07] ****	*Liche*	NA
	Aboveground biomass	**↑ 0.32 [0.12, 0.52] ****	*Aboveground biomass*	NA
	Community height	**↑ 1.04 [0.79, 1.30] *****	*Community height*	NA
**Microbial community**	*Bacterial Biomass*	↓ -0.38 [-0.94, 0.18]	*Bacterial Biomass*	↓ - 1817599 [-4177364, 542166]
	Fungal Biomass	↑ 0.12 [-0.40, 0.64]	*Fungal Biomass*	↑ 169005 [-189929, 527940]
	FB-Ratio	↑ 0.45 [-0.08, 0.97] (*)	***FB-Ratio***	**↑ 0.03 [0.00, 0.06] ***

[1]Significance levels: **Significant results with *p*<0.05 are highlighted in bold.* *p*<0.05; ** <0.01; *** <0.001**. Trends (*p*<0.1) are indicated with (*).

From left to right: The type of environmental driver ('Type') and the specific driver ('Driver') investigated, the effect of the warming treatment on the driver (i.e. meta-analysis results: 'Slope') for two effect sizes: Hedges Standardized Mean Difference (*SMD*, left) vs. *R*aw Mean Difference (*RMD*, right). Model results are presented as meta-analysis slopes and 95% confidence intervals between square brackets, as well as significance levels based on *p*-values. Significant drivers and results are highlighted in **bold**. An upward (↑) or downward (↓) arrow before the model estimates indicates that the warming treatment increased or decreased the environmental driver. Sample sizes can be found in **Supp. Table 5** ('Context-dependencies'). Note that we do not include raw mean differences models for the vegetation community ('NA') because the methodologies across experiments to measure the %cover, biomass, or plant height differed too much, so comparing raw values is not warranted.

Supp. Table 4: Mean effect sizes (*Hedges SMD*), 95% confidence intervals, experimental warming durations, and mean percentage change, for the 136 datasets used in the meta-analysis testing warming effects on ecosystem respiration [*ER*].

Exp ID	Dataset ID	*Hedges SMD*	Lower CI	Upper CI	Duration	*ROM*	Percentage change (%)
ALA_1	ALA_1_2009	0.606	0.508	0.704	1	0.210	23.4
	ALA_1_2010	0.333	0.242	0.424	2	0.114	12.0
	ALA_1_2011	0.349	0.270	0.428	3	0.118	12.6
	ALA_1_2012	0.457	0.350	0.564	4	0.147	15.8
	ALA_1_2013	0.325	0.221	0.429	5	0.127	13.6
	ALA_1_2014	0.196	0.092	0.301	6	0.071	7.3
	ALA_1_2015	0.128	0.019	0.236	7	0.053	5.5
	**ALA_1_2016**	**-0.077**	**-0.195**	**0.040**	**8**	**-0.031**	**-3.1**
	**ALA_1_2017**	**-0.205**	**-0.321**	**-0.089**	**9**	**-0.084**	**-8.1**
	**ALA_1_2018**	**-0.127**	**-0.254**	**0.000**	**10**	**-0.057**	**-5.6**
	**ALA_1_2019**	**-0.168**	**-0.375**	**0.040**	**11**	**-0.065**	**-6.3**
ALA_2	ALA_2_2015	0.634	0.292	0.976	0	0.311	36.4
ALA_3	ALA_3_2016	0.011	-0.652	0.674	0	0.005	0.5
ALA_4	ALA_4_2017	0.784	0.163	1.404	1	0.399	49.1
	ALA_4_2019	0.679	0.340	1.019	3	0.399	49.0
AUS_1	AUS_1_2016	2.066	1.194	2.938	2	1.009	174.3
	AUS_1_2017	1.162	0.103	2.222	3	0.324	38.2
	AUS_1_2018	0.672	-0.040	1.385	4	0.194	21.4
CAN_2	CAN_2_2011	0.193	-0.228	0.615	0	0.066	6.8
	CAN_2_2012	1.154	0.800	1.508	1	0.379	46.1
	CAN_2_2013	0.374	0.023	0.726	2	0.120	12.7
CAN_3	CAN_3_2011	0.706	0.218	1.194	0	0.258	29.4
	CAN_3_2012	0.572	0.232	0.911	1	0.235	26.5
	CAN_3_2013	0.396	0.028	0.763	2	0.182	20.0
CAN_4	CAN_4_2011	0.717	0.253	1.181	0	0.284	32.8
	CAN_4_2012	0.573	0.250	0.897	1	0.257	29.3
	CAN_4_2013	0.743	0.347	1.139	2	0.245	27.8
CAN_5	CAN_5_2018	0.150	-0.832	1.131	6	0.055	5.7
	CAN_5_2019	0.388	-0.388	1.164	7	0.134	14.4
CHI_1	**CHI_1_2019**	**-0.799**	**-1.631**	**0.032**	**5**	**-0.204**	**-18.4**
CHI_1	**CHI_1_2020**	**-0.515**	**-1.328**	**0.299**	**6**	**-0.123**	**-11.6**
CHI_2	CHI_2_2019	0.950	-0.084	1.984	4	0.462	58.7
	CHI_2_2020	0.993	0.145	1.841	5	0.482	62.0
CHI_3	CHI_3_2019	1.027	-0.016	2.070	5	0.220	24.6
	CHI_3_2020	0.753	-0.075	1.581	6	0.450	56.9
CHI_4	CHI_4_2014	0.250	-0.063	0.563	1	0.120	12.7
	CHI_4_2015	0.440	0.125	0.755	2	0.202	22.4
	**CHI_4_2016**	**-0.033**	**-0.327**	**0.261**	**3**	**-0.012**	**-1.2**
FIN_1	FIN_1_2013	0.664	0.132	1.195	19	0.299	34.9
GRE_1	GRE_1_2013	0.676	0.279	1.073	1	0.302	35.3

	GRE_1_2014	0.762	0.403	1.121	2	0.321	37.9
GRE_2	GRE_2_2014	0.475	0.033	0.916	1	0.261	29.9
GRE_4	GRE_4_2012	0.691	-0.012	1.394	5	0.308	36.1
	GRE_4_2015	0.235	-0.403	0.873	8	0.127	13.5
GRE_5	GRE_5_2012	0.544	-0.021	1.108	5	0.269	30.9
	GRE_5_2015	0.408	-0.272	1.087	8	0.214	23.9
GRE_6	GRE_6_2008	1.095	0.725	1.466	1	0.345	41.1
	GRE_6_2009	0.336	-0.024	0.697	2	0.178	19.5
	GRE_6_2010	0.719	0.374	1.065	3	0.247	28.0
	GRE_6_2011	1.174	0.741	1.607	4	0.336	39.9
	GRE_6_2012	0.451	0.109	0.793	5	0.150	16.2
	GRE_6_2013	0.219	-0.142	0.579	6	0.093	9.7
	**GRE_6_2014**	**-0.101**	**-0.456**	**0.255**	**7**	**-0.044**	**-4.3**
	**GRE_6_2015**	**-0.262**	**-0.664**	**0.140**	**8**	**-0.130**	**-12.2**
	**GRE_6_2016**	**-0.265**	**-0.595**	**0.064**	**9**	**-0.116**	**-11.0**
	**GRE_6_2017**	**-0.580**	**-0.954**	**-0.205**	**10**	**-0.222**	**-19.9**
	**GRE_6_2018**	**-0.301**	**-0.658**	**0.056**	**11**	**-0.161**	**-14.8**
	**GRE_6_2019**	**-0.261**	**-0.631**	**0.110**	**12**	**-0.119**	**-11.2**
	**GRE_6_2020**	**-0.043**	**-0.428**	**0.341**	**13**	**-0.016**	**-1.6**
ICE_1	**ICE_1_2007**	**-0.169**	**-1.303**	**0.964**	**10**	**-0.097**	**-9.3**
ICE_1	ICE_1_2008	0.235	-0.420	0.891	11	0.169	18.4
ITA_1	ITA_1_2020	1.545	0.839	2.251	12	0.353	42.3
ITA_2	ITA_2_2020	0.269	-0.354	0.892	12	0.065	6.8
ITA_3	ITA_3_2020	1.047	0.061	2.032	8	0.261	29.8
ITA_4	ITA_4_2020	1.129	0.104	2.154	8	0.237	26.7
ITA_5	ITA_5_2020	0.113	-0.603	0.830	2	0.026	2.7
NOR_1	NOR_1_2011	0.743	0.455	1.031	1	0.489	63.0
NOR_1	NOR_1_2012	0.425	0.033	0.818	2	0.308	36.0
NOR_6	NOR_6_2020	1.437	0.658	2.215	3	0.504	65.6
NOR_7	NOR_7_2020	0.285	-0.488	1.058	3	0.153	16.5
NOR_8	NOR_8_2020	1.591	0.673	2.509	3	0.824	128.0
NOR_9	NOR_9_2020	0.950	0.168	1.731	2	0.595	81.3
NOR_10	NOR_10_2020	0.593	-0.151	1.337	2	0.409	50.5
NOR_11	**NOR_11_2020**	**-0.098**	**-0.814**	**0.618**	**2**	**-0.039**	**-3.8**
NOR_12	**NOR_12_2020**	**-0.082**	**-0.867**	**0.703**	**2**	**-0.046**	**-4.5**
RUS_1	RUS_1_2012	0.603	0.120	1.085	0	0.238	26.9
	RUS_1_2013	0.307	-0.108	0.723	1	0.136	14.5
	RUS_1_2014	1.144	0.198	2.090	2	0.380	46.2
RUS_2	RUS_2_2012	0.308	-0.214	0.831	0	0.121	12.8
	RUS_2_2013	0.165	-0.249	0.578	1	0.084	8.7
	RUS_2_2014	0.218	-0.737	1.173	2	0.121	12.9
RUS_3	RUS_3_2012	1.186	0.623	1.749	0	0.408	50.4
	RUS_3_2013	0.649	0.217	1.080	1	0.230	25.9
	RUS_3_2014	0.198	-1.005	1.401	2	0.089	9.3
SVA_1	SVA_1_2016	0.695	0.235	1.155	0	0.390	47.6
	SVA_1_2017	1.251	0.775	1.728	1	0.793	121.0

SVA_2	SVA_2_2016	0.554	0.107	1.000	0	0.528	69.5
SVA_2	SVA_2_2017	0.754	0.309	1.199	1	0.450	56.9
SVA_3	SVA_3_2016	0.507	0.031	0.983	0	0.266	30.5
	SVA_3_2017	0.409	-0.061	0.879	1	0.225	25.2
SVA_4	SVA_4_2018	1.301	0.581	2.020	2	1.014	175.6
	SVA_4_2019	1.401	0.662	2.141	3	0.780	118.0
SVA_5	SVA_5_2003	0.424	-0.511	1.358	0	0.265	30.3
	SVA_5_2004	0.398	-0.325	1.120	1	0.293	34.1
	SVA_5_2005	0.635	0.067	1.203	2	0.245	27.7
	SVA_5_2007	0.236	-0.482	0.954	4	0.127	13.6
SVA_6	SVA_6_2003	0.123	-0.754	1.001	0	0.061	6.3
	SVA_6_2004	0.189	-0.529	0.906	1	0.127	13.5
	SVA_6_2005	0.194	-0.368	0.755	2	0.105	11.1
	**SVA_6_2007**	**-0.039**	**-0.659**	**0.581**	**4**	**-0.021**	**-2.0**
SVA_7	SVA_7_2018	0.752	-0.210	1.713	16	0.371	45.0
SVA_8	SVA_8_2018	1.809	0.316	3.302	16	1.102	201.1
SVA_9	SVA_9_2018	0.611	-0.364	1.585	16	0.476	60.9
SWE_1	SWE_1_2008	0.413	-0.147	0.973	13	0.213	23.7
	SWE_1_2017	0.785	0.210	1.360	22	0.373	45.2
	SWE_1_2018	0.573	0.053	1.094	23	0.272	31.2
	SWE_1_2019	0.934	-0.372	2.239	24	0.280	32.3
SWE_2	SWE_2_2008	0.756	0.039	1.474	13	0.288	33.3
	SWE_2_2017	1.069	0.464	1.673	22	0.397	48.8
	SWE_2_2018	1.720	0.994	2.445	23	0.818	126.5
	SWE_2_2019	1.575	-0.011	3.161	24	0.632	88.1
SWE_3	SWE_3_2008	0.531	-0.039	1.101	14	0.274	31.6
	SWE_3_2017	1.115	0.571	1.659	23	0.380	46.3
	SWE_3_2018	0.916	0.333	1.498	24	0.511	66.8
	SWE_3_2019	1.495	0.093	2.898	25	0.377	45.7
SWE_4	SWE_4_2008	0.899	0.312	1.487	14	0.455	57.6
	SWE_4_2017	1.114	0.478	1.749	23	0.402	49.4
	SWE_4_2018	1.001	0.413	1.589	24	0.628	87.4
	SWE_4_2019	1.054	-0.269	2.377	25	0.290	33.7
SWE_5	SWE_5_2008	0.467	-0.205	1.139	14	0.287	33.2
	SWE_5_2017	0.226	-0.492	0.944	23	0.130	13.9
	SWE_5_2018	0.317	-0.197	0.830	24	0.151	16.4
	SWE_5_2019	0.290	-1.103	1.683	25	0.087	9.1
SWE_6	SWE_6_2006	1.252	0.538	1.967	7	0.365	44.0
	SWE_6_2007	1.100	0.466	1.734	8	0.674	96.2
	SWE_6_2015	1.349	0.875	1.823	16	0.447	56.3
SWE_7	SWE_7_2011	0.812	0.485	1.138	22	0.414	51.2
SWE_12	SWE_12_2001	1.195	0.686	1.703	3	0.390	47.6
	**SWE_12_2018**	**-0.927**	**-1.506**	**-0.348**	**20**	**-0.546**	**-42.1**
SWE_14	SWE_14_2001	0.659	0.139	1.178	3	0.260	29.7
SWE_15	**SWE_15_2001**	**-0.968**	**-1.724**	**-0.211**	**3**	**-0.482**	**-38.3**
	**SWE_15_2018**	**-0.783**	**-1.380**	**-0.187**	**20**	**-0.436**	**-35.3**

SWE_16	SWE_16_2003	1.056	0.368	1.744	3	0.652	92.0
	SWE_16_2005	0.228	-0.328	0.784	5	0.108	11.4
	SWE_16_2006	0.524	0.048	1.001	6	0.272	31.2
	SWE_16_2007	0.803	0.159	1.448	7	0.366	44.2
**MEAN**	**136 datasets**	**0.568**	**0.441**	**0.696**		**0.260**	**29.8**

From left to right, the columns show alphabetically + chronologically ordered experimental IDs (‘Exp ID’), dataset IDs (‘Dataset ID’, i.e. Exp ID followed by the *ER* measurement year), *ER* ‘Hedges Standardized Mean Differences or *SMD*s as effect sizes, lower and upper boundaries of the 95% confidence intervals (‘lower CI, ‘upper CI’), experimental warming duration (‘Duration’), mean log-transformed ratio of means (‘*ROM*’), and mean percentage change (‘Percentage change’, calculated as 100*(exp(*ROM*)-1)). Negative effect sizes, implying *ER* decreases with experimental warming, are highlighted in **bold**. The final row presents the mean pooled model estimate + confidence intervals of the meta-analysis across the 136 datasets.

Supp. Table 5: Sample sizes across the meta-regression models.

Model	Type	Driver	Nr (%) DS	Nr (%) Exp
**1) Indirect warming effects** (Treatment Effect sizes)	**Climate**	*SMD* Air temperature	77 (57%)	36 (64%)
		SMD Soil temperature	118 (87%)	54 (96%)
		SMD Soil moisture	111 (82%)	41 (73%)
	**Soil**	*SMD* SOM (mineral, organic)	28, 41 (21, 30%)	10, 16 (18, 29%)
		SMD SOC (mineral, organic)	42, 65 (31, 48%)	11, 21 (20, 38%)
		SMD SON (mineral, organic)	42, 65 (31, 48%)	11, 21 (20, 38%)
		SMD CN (mineral, organic)	38, 71 (28, 52%)	10, 24 (18, 43%)
		SMD pH (mineral, organic)	27, 53 (20, 39%)	9, 24 (16, 43%)
		SMD Bulk density (mineral, organic)	31, 49 (23, 36%)	7, 15 (12, 27%)
		SMD Organic layer depth	44 (32%)	18 (32%)
	**Vegetation**	*SMD* Graminoids	100 (74%)	41 (73%)
		SMD Forbs	111 (82%)	46 (82%)
		SMD Deciduous shrubs	90 (66%)	39 (70%)
		SMD Evergreen shrubs	88 (65%)	33 (59%)
		SMD Mosses	88 (65%)	41 (73%)
		SMD Lichens	78 (57%)	35 (62%)
		SMD Biomass	61 (45%)	20 (36%)
		SMD Community height	43 (32%)	23 (41%)
	**Microbial**	*SMD* Bacterial Biomass	16 (12%)	8 (14%)
		SMD Fungal Biomass	16 (12%)	8 (14%)
		SMD FB-Ratio	16 (12%)	8 (14%)
**2) Context-dependencies** (CTL plot means)	**Climate**	Zone	136 (100%)	56 (100%)
		Permafrost probability	133 (98%)	55 (98%)
		Air temperature	77 (57%)	36 (64%)
		Soil temperature	118 (87%)	54 (96%)
		Soil moisture	111 (82%)	41 (73%)
		Soil moisture class	136 (100%)	56 (100%)
	**Soil**	SOM (mineral, organic)	28, 41 (21, 30%)	10, 16 (18, 29%)
		Soil Carbon stock	131 (96%)	51 (91%)
		TC (mineral, organic)	42, 70 (31, 51%)	11, 25, (20, 45%)
		TN (mineral, organic)	43, 70 (32, 51%)	10, 27 (18, 48%)
		CN (mineral, organic)	39, 75 (29, 55%)	10, 24 (18, 43%)
		pH class	136 (100%)	56 (100%)
		pH (mineral, organic)	29, 55 (21, 40%)	10, 24 (18, 43%)
		Bulk density (mineral, organic)	38, 69 (28, 51%)	9, 22 (16, 39%)
		Organic layer depth	78 (57%)	28 (50%)
	**Vegetation**	Vegetation class	136 (100%)	56 (100%)
		Net primary productivity	136 (100%)	56 (100%)

From left to right: The model (**Ext. Table 3-4)**, and the type of environmental driver investigated (‘Type’) across the meta-regressions, the specific driver used as predictor to test the effect on *ER* response rates (‘Driver’), followed by sample sizes for each driver i.e. number (and percentage between brackets) of datasets and experiments (‘Nr

(%) DS'; 'Nr (%) Exp').

Supp. Figures

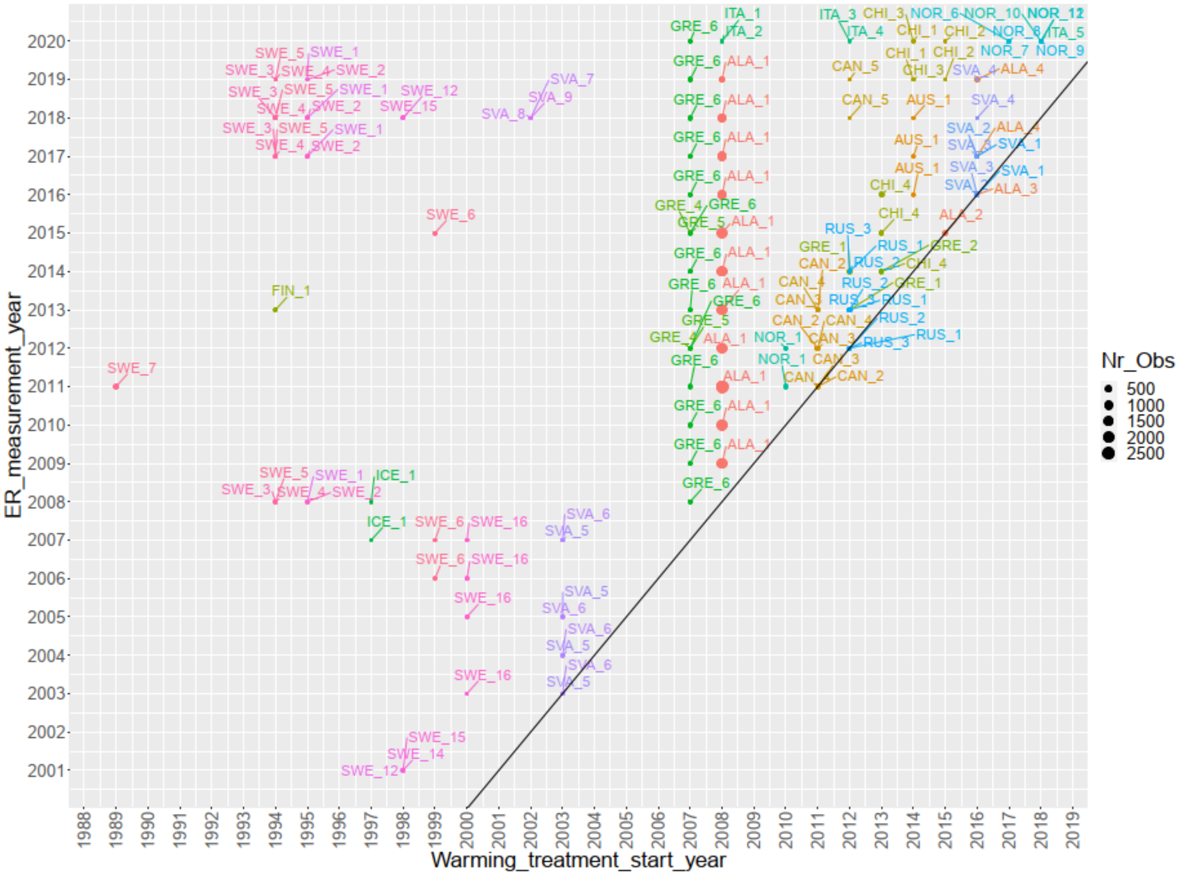

Supp. Figure 1: Overview of the timing of the warming experiments and the *ER* measurements across the 136 datasets. The start year of the experimental warming treatment is displayed on the x-axis, while the year of *ER* measurements is displayed on the y-axis. Every dataset is represented by a colored dot with the color representing a unique warming experiment (e.g. GRE_6 = dark green) and the size representing the number of *ER* measurements or observations for that year, which are averaged for the analyses. The black line represents occasions where the *ER* measurements were done in the first growing season of experimental warming (y=x: start year of warming treatment = *ER* measurement year). See **Supp. Fig. 2** for a detailed overview of the distribution of the measurements across the growing season.

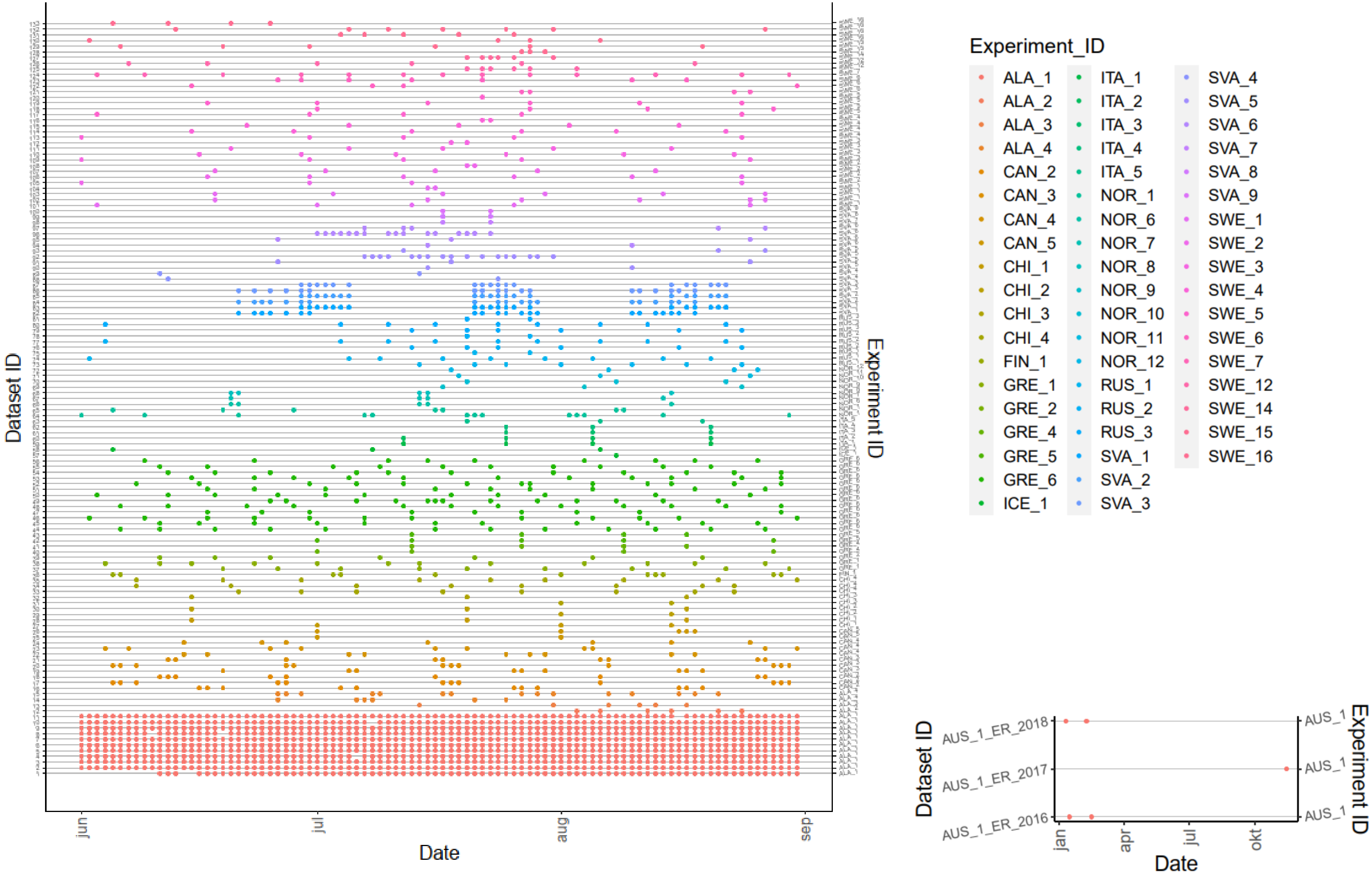
Dataset ID
Experiment ID
jun
jul
aug
sep
Date
Experiment_ID
ALA_1
ALA_2
ALA_3
ALA_4
CAN_2
CAN_3
CAN_4
CAN_5
CHI_1
CHI_2
CHI_3
CHI_4
FIN_1
GRE_1
GRE_2
GRE_4
GRE_5
GRE_6
ICE_1
ITA_1
ITA_2
ITA_3
ITA_4
ITA_5
NOR_1
NOR_6
NOR_7
NOR_8
NOR_9
NOR_10
NOR_11
NOR_12
RUS_1
RUS_2
RUS_3
SVA_1
SVA_2
SVA_3
SVA_4
SVA_5
SVA_6
SVA_7
SVA_8
SVA_9
SWE_1
SWE_2
SWE_3
SWE_4
SWE_5
SWE_6
SWE_7
SWE_12
SWE_14
SWE_15
SWE_16
Dataset ID
AUS_1_ER_2018
AUS_1_ER_2017
AUS_1_ER_2016
jan
apr
jul
okt
Date
AUS_1
AUS_1
AUS_1
Experiment ID

Supp. Figure 2: Overview of the timing of the *ER* measurement dates (x-axis) across the growing season for each of the 136 datasets (y-axis). The left figure displays measurement dates for all Northern hemisphere datasets, where the growing season was set between June-September. The right figure displays measurement dates for the single Southern hemisphere dataset from Australia i.e. AUS_1, where the growing season was set between October-February. Every measurement date is represented by a circle with the color representing a unique warming experiment (e.g. GRE_6 = dark green).

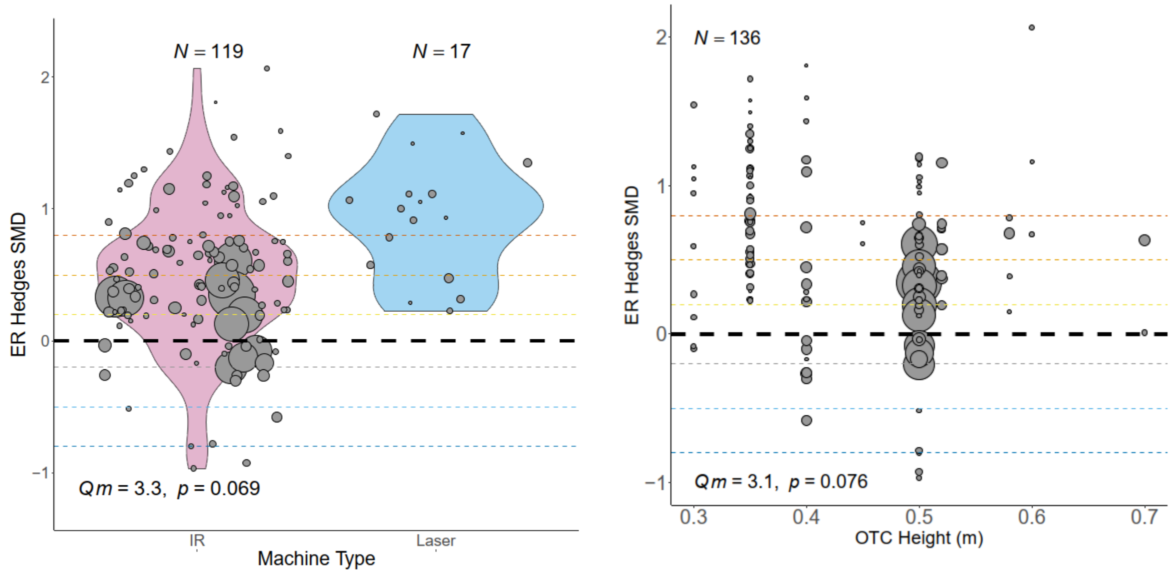

Supp. Figure 3: Trends (*p*<0.1) in the effects of flux measurement machine type (a) and OTC height (b) on *ER* response rates. Machine type represents whether *ER* measurements were taken with an infrared 'IR' or 'laser' machine. OTC height represents the height of the open-top-chambers used for experimental warming. *ER* Hedges Standardized Mean Differences (SMDs) for individual datasets are displayed with grey bubbles, calculated as [mean *ER* of the warmed plots - mean *ER* of the control plots] / pooled standard deviation). Bubble size denotes the weight of the observation used in the meta-regression models quantified as the inverse of the square root of within-study variance, with greater bubbles indicating greater weights. In (a), violin plots of the actual data across the two factor levels are displayed, i.e., kernel density estimation of the underlying distributions. Within the violin plots, meta-regression model estimates and 95% confidence intervals are displayed with yellow circles and error bars. In (b), the significant regression lines with 95%CI are displayed with black lines and grey shaded areas, respectively. Top left in each panel shows the sample size ('N', number of datasets), and bottom left shows the '*Qm*' (Q-value of importance of the environmental drivers) and '*p*'-value of the meta-regression models. Dotted horizontal lines (y=0.2, 0.5, 0.8 and -0.2, -0.5, -0.8) reflect small, medium, and large positive and negative Hedges SMD effect sizes, respectively[12] or subsequently greater *ER* increases and decreases with warming.

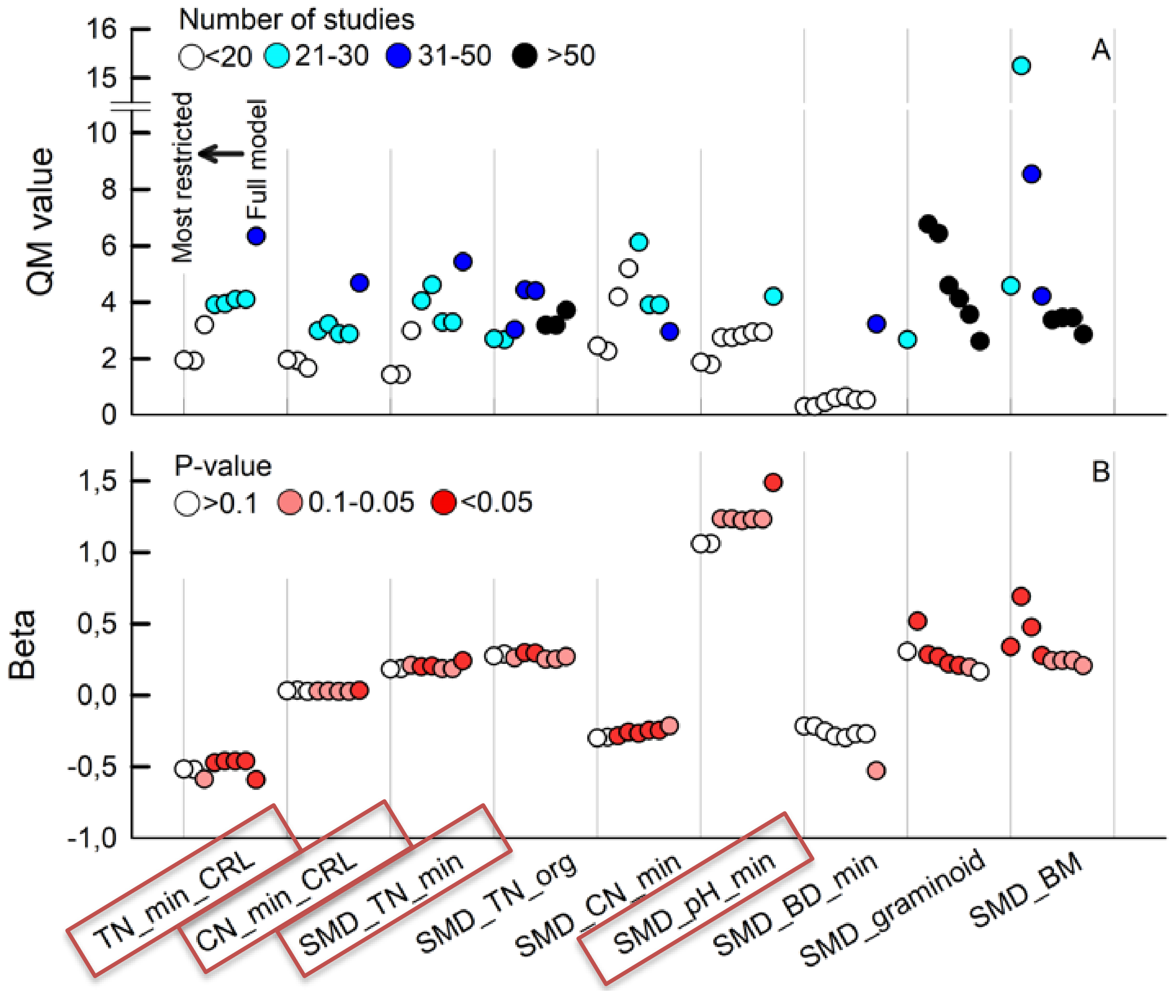

Supp. Figure 4: Results of the sensitivity analysis on meta-regression models for drivers showing significant effects on *ER* Hedges SMD, and those showing trends in the latter. Significant environmental drivers based on the full model are presented on the x-axis with a red frame, trends without a frame. Different dots per driver represent different restrictive sample size scenarios, increasing in restrictiveness from right to left: the <u>full model</u> *most right*, followed by <u>6 scenarios where calendar year differences</u> between *ER* measurements and driver measurements are restricted to maximum 5, 4, 3, 2, 1, 0 years, and the *most left* scenario represents <u>no replication of driver measurements</u>, including driver measurement only for the *ER* year closest to the driver year). Panel A shows the *Qm*-value per model per scenario, which represents the explanatory power of each driver, while Panel B represents the beta or (meta-)regression parameter for each driver. Dots are colored by sample size (nr. of datasets) in panel A, and by *p*-value of the meta-regression model in panel B.TN, CN, SMD, BD, and BM reflect total nitrogen, carbon:nitrogen ratio, Hedges Standardized Mean Differences, bulk density, and aboveground biomass.

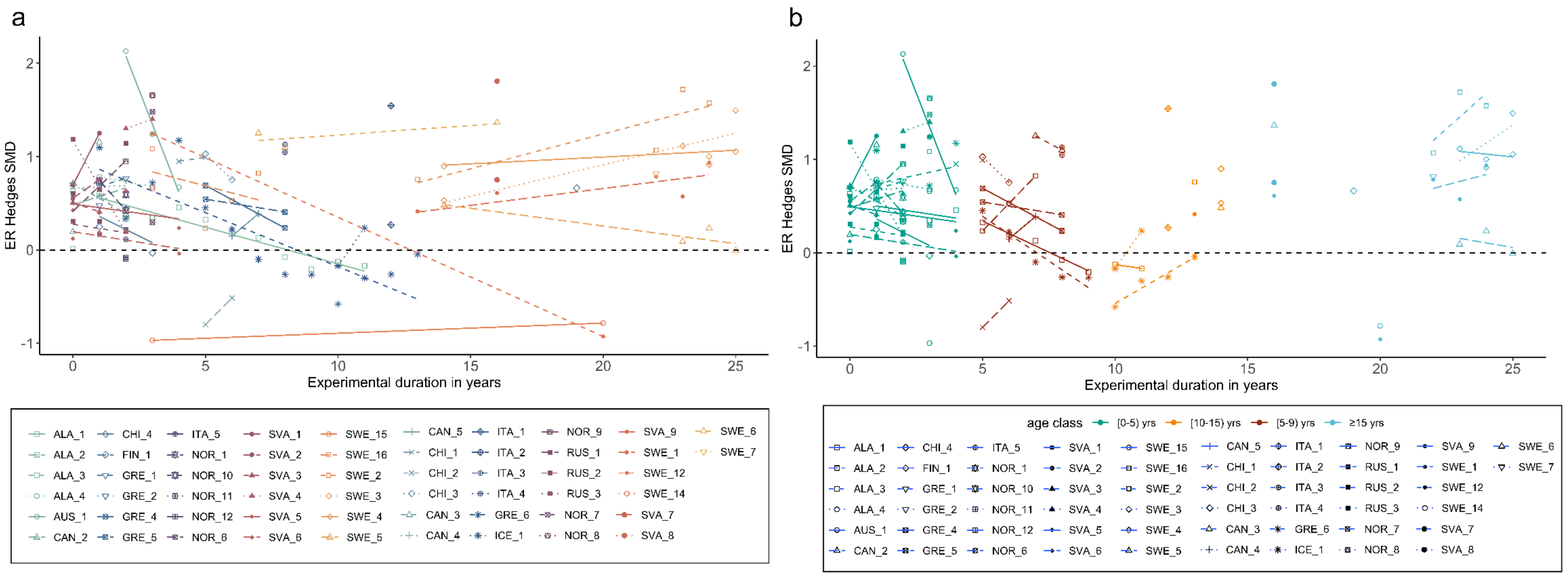

Supp. Figure 5: Temporal patterns in *ER* response rates to experimental warming duration *within* experiments, across (a) and within (b) duration age classes. Linear regression lines of *ER* response against experimental warming duration within each experiment are shown. In **a**, distinct combinations of symbols, colors, and line types represent *ER* Hedges SMD for individual datasets and their corresponding regression lines *across* age classes. In **b**, *ER* Hedges SMD for individual datasets and their regression lines of *ER* response rates to experimental warming duration *within* age classes ([0-5) years, [5-10) years, [10-15) years, ≥15 years) are shown, using colors to distinguish between the four age classes.

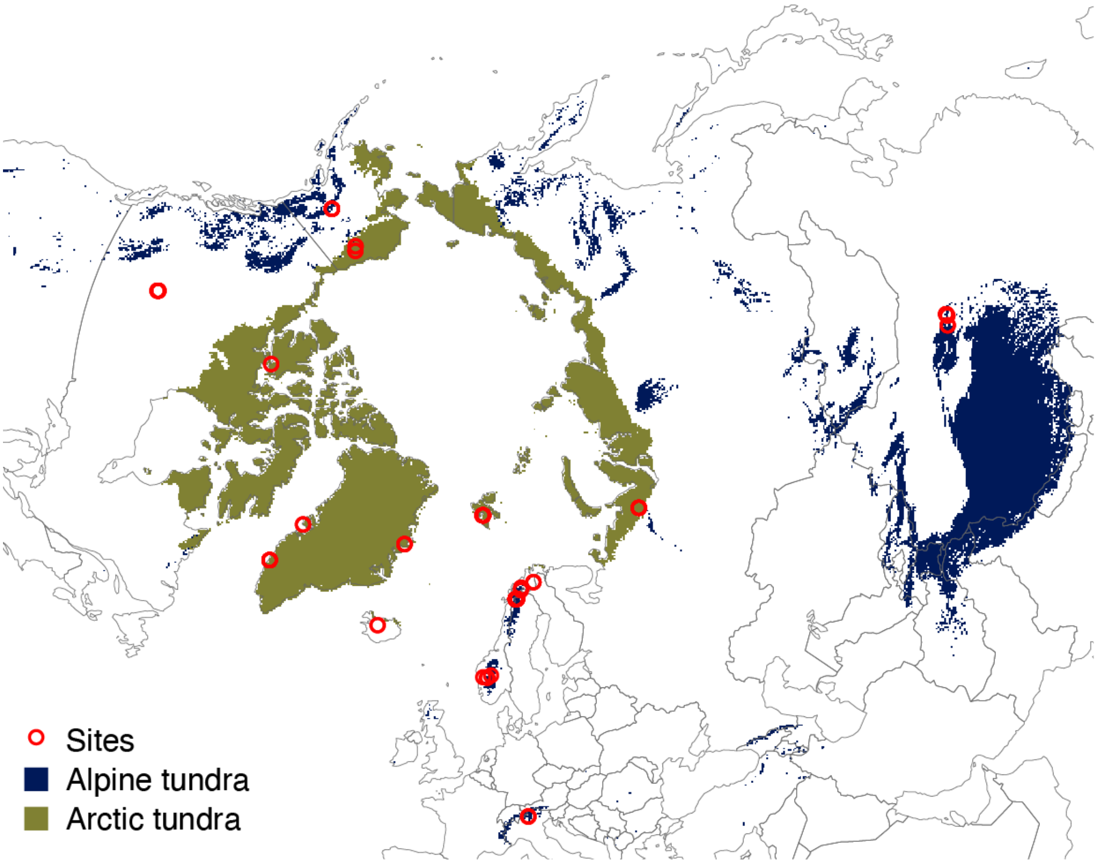

Supp. Figure 6: Map of the areas of arctic tundra (green) and circumarctic alpine tundra including the Tibetan plateau (blue) used for upscaling, relative to the location of study sites included in the dataset (red circles).

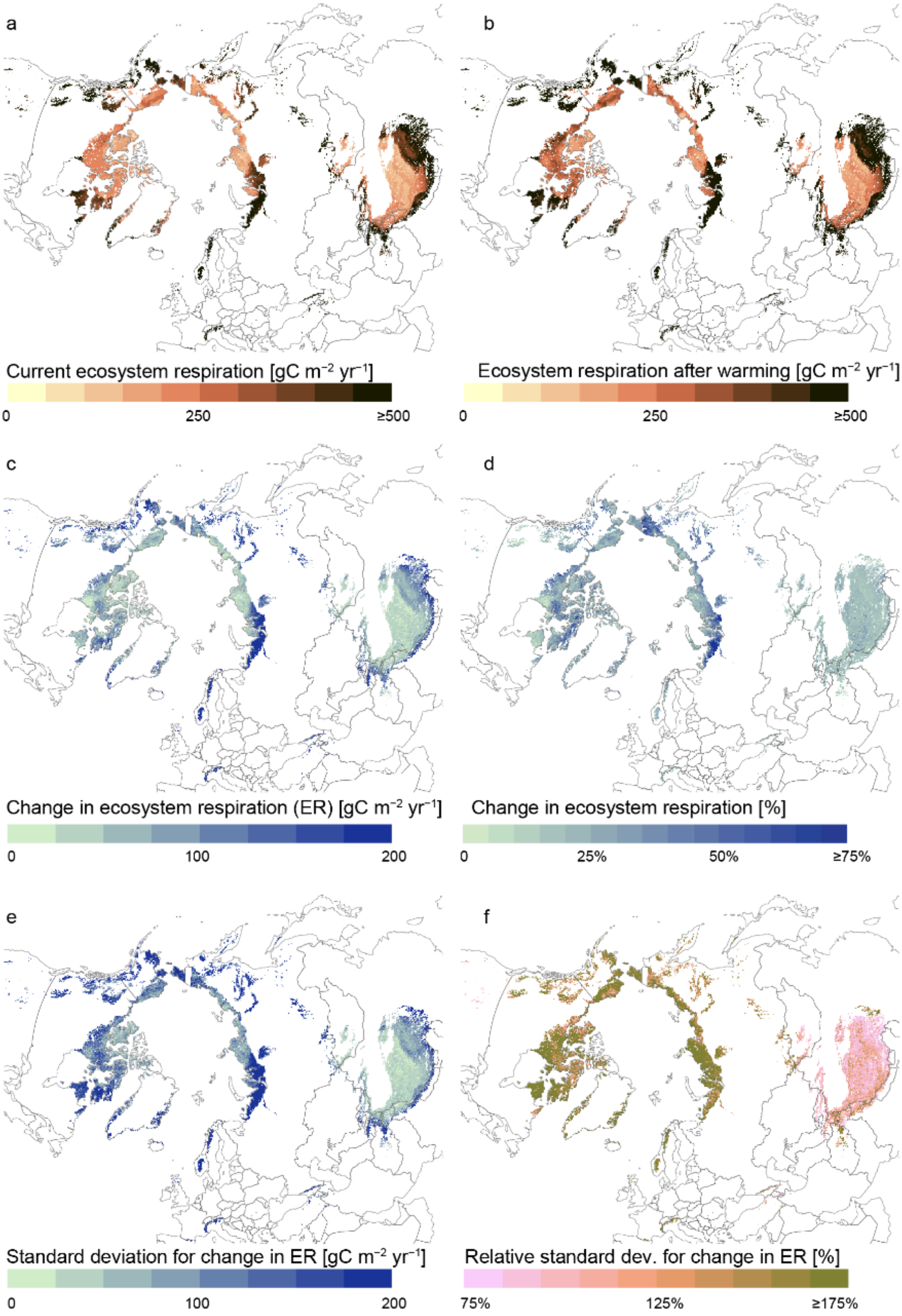
a
b
Current ecosystem respiration [gC m⁻² yr⁻¹]
0
250
≥500
Ecosystem respiration after warming [gC m⁻² yr⁻¹]
0
250
≥500
c
d
Change in ecosystem respiration (ER) [gC m⁻² yr⁻¹]
0
100
200
Change in ecosystem respiration [%]
0
25%
50%
≥75%
e
f
Standard deviation for change in ER [gC m⁻² yr⁻¹]
0
100
200
Relative standard dev. for change in ER [%]
75%
125%
≥175%

Supp. Figure 7: Spatial patterns of current ecosystem respiration (*ER*) (a) and its responses to 1.4°C warming **(b-d)** across the arctic and circumarctic alpine tundra, with uncertainty estimates **(e, f)**. Current *ER* (a) and *ER* after 1.4°C warming **(b)**, i.e. the average observed warming in the open-top chamber (OTC) warming experiments in our database, were compared to calculate absolute (c) and relative (d) changes in *ER*. Uncertainty estimates are given as standard error for the absolute change in *ER* (e) and as relative standard error for *ER* (standard error compared to the change in *ER*) (f). To conduct the upscaling, we used the significant meta-regression multi-factor model based on the warming experiments, i.e. including the ratio of means (ROM) as effect size (response) and TN and C:N-ratio in the mineral layer (Qm=6.7, p<0.05, N=39) as significant explanatory drivers , and applied this model to each 1 km x 1 km grid cell using global gridded soil data for TN concentration and C:N-ratio of the mineral layer, resulting in a relative change in respiration. We also multiplied this relative change to spatially explicit baseline *ER*, calculated from spatially explicit global soil respiration and plant respiration databases, to obtain absolute changes in *ER*. For the whole region, *ER* increased from 3.4 to 4.3 PgC yr-1 (increase of 0.86 PgC yr-1 with std. deviation of 1.36 PgC yr-1) or by 25% (std. deviation = 40%). See Methods section and **Supp. Methods 3** for more information.

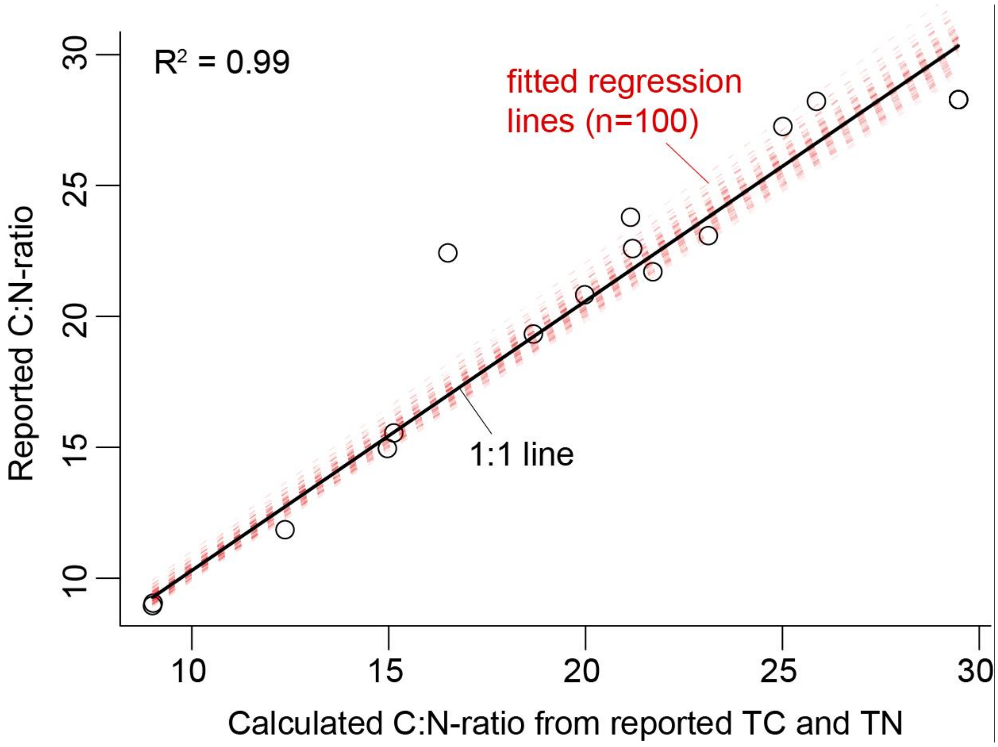

Supp. Figure 8: Relationship between reported measurement-based C:N values (y-axis) and calculated C:N values based on reported TN and TC values. The red dotted lines show the fitted (n=100) regressions used to estimate the uncertainty of deriving the C:N from the separately reported TN and TC.

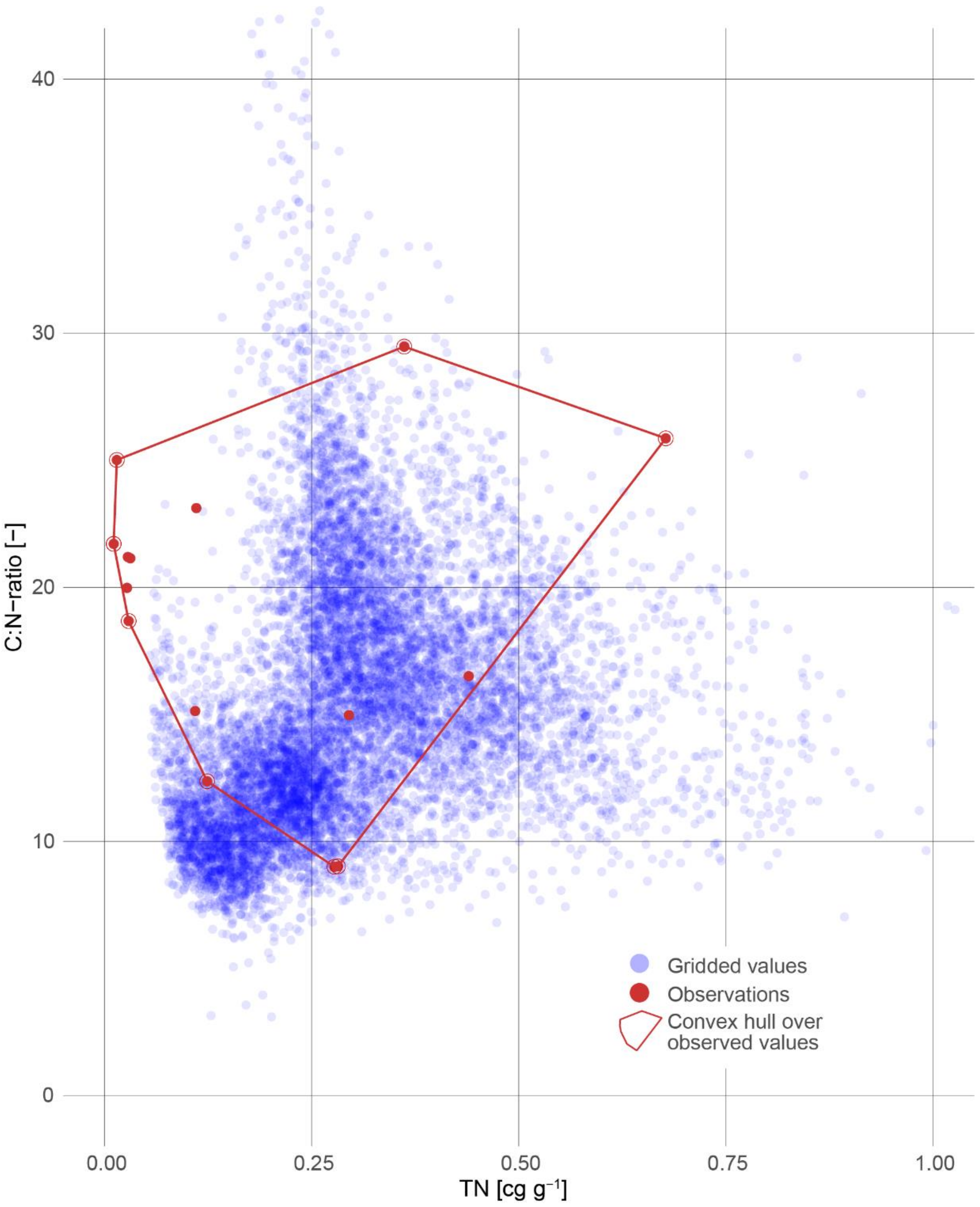

Supp. Figure 9: Comparison of observed TN and C:N-ratio (red, based on our experimental dataset) and gridded mean TN and C:N-ratio (blue, from ISRIC soil database) used for upscaling. From the ISRIC soil database, we sampled randomly 0.1% from the ca 10 million gridded datapoints within the study area. The convex hull shows the areas which the observations cover, ca 64% of the gridded values.

References

1. Jeanbille, M. *et al.* Site-specific responses of fungal and bacterial abundances to experimental warming in litter and soil across Arctic and alpine tundra. *Arct. Sci.* **14**, 1–14 (2021).
2. Walker, D. A. *et al.* The Circumpolar Arctic vegetation map. *J. Veg. Sci.* **16**, 267–282 (2005).
3. Obu, J. *et al.* Northern Hemisphere permafrost map based on TTOP modelling for 2000–2016 at 1 km2 scale. *Earth-Science Rev.* **193**, 299–316 (2019).
4. FAO & ITPS. *Global Soil Organic Carbon Map (GSOCmap)*. *Technical Report.* (2018) doi:10.4060/ca7597en.
5. Hugelius, G. *et al.* A new data set for estimating organic carbon storage to 3 m depth in soils of the northern circumpolar permafrost region. *Earth Syst. Sci. Data* **5**, 393–402 (2013).
6. Running, S. & Zhao, M. MOD17A3HGF MODIS/Terra Net Primary Production Gap-Filled Yearly L4 Global 500 m SIN Grid V006 [Data set]. NASA EOSDIS Land Processes DAAC. (2019).
7. Davidson, E. A. & Janssens, I. A. Temperature sensitivity of soil carbon decomposition and feedbacks to climate change. *Nature* **440**, 165–173 (2006).
8. Luan, J., Liu, S., Wang, J. & Zhu, X. Factors Affecting Spatial Variation of Annual Apparent Q10 of Soil Respiration in Two Warm Temperate Forests. *PLoS One* **8**, 1–8 (2013).
9. Wu, Q., Ye, R., Bridgham, S. D. & Jin, Q. Limitations of the Q10 Coefficient for Quantifying Temperature Sensitivity of Anaerobic Organic Matter Decomposition: A Modeling Based Assessment. *J. Geophys. Res. Biogeosciences* **126**, 1–18 (2021).
10. Perkins, D. M. *et al.* Consistent temperature dependence of respiration across ecosystems contrasting in thermal history. *Glob. Chang. Biol.* **18**, 1300–1311 (2012).
11. Epron, D. Separating autotrophic and heterotrophic components of soil respiration: Lessons learned from trenching and related root-exclusion experiments. *Soil Carbon Dyn. An Integr. Methodol.* 157–168 (2010) doi:10.1017/CBO9780511711794.009.
12. Cohen, J. *Statistical power analysis for the behavioral sciences*. (Routledge, 2013).
